# Small extracellular vesicles from the human endothelial cell line EA.hy 926 exert a self-cell activation and modulate DENV-2 genome replication and infection in naïve endothelial cells

**DOI:** 10.1371/journal.pone.0310735

**Published:** 2024-09-26

**Authors:** María-Angélica Calderón-Peláez, L. Johana Madroñero, Jaime E. Castellanos, Myriam L. Velandia-Romero

**Affiliations:** Virology group, Vice-chancellor of research, Universidad El Bosque, Bogotá, Colombia; Telethon Institute of Genetics and Medicine, ITALY

## Abstract

Extracellular vesicles (EVs) play crucial roles in cell signaling and communication, transporting molecules that convey a message to target cells. During infectious diseases, EVs can also carry viral molecules that may contribute to viral spread, as previously reported for dengue virus (DENV). EVs from infected endothelial cells (EC) may harbor viral segments and various sets of molecules that could contribute to endothelial dysfunction during severe dengue. However, the effect of these EVs on non-infected EC (NIC) remain unknown. We characterized the EVs produced by the human EC line EA.hy 926 infected with DENV-2 and assessed their functional impact on polarized NIC. Results showed that infection induced an increased in the quantity of produced EVs, which differentially carried proteins mainly involved in proteosome activity, along with a peptide of the NS5 viral protein. Additionally, all types of Y-RNAs were found, accompanied by a set of differentially loaded microRNAs (miRs) that could regulate DENV genome. Pre-treatment of polarized NIC with small EVs (_s_EVs) from infected EC before DENV-2 infection caused EC activation, a decrease in viral genome replication, and a protective effect against barrier disruption during the first 24h post-infection, suggesting that _s_EVs could be important in the pathology or resolution of DENV and a promising therapeutic tool for infectious diseases.

## Introduction

Cell communication strategies include soluble mediators like cytokines, chemokines [1], as well as various types of vesicles [2]. Extracellular vesicles (EVs) are spherical particles of different sizes (30–5000 nm), surrounded by a lipidic membrane. Importantly, EVs carry DNA, RNA, and small noncoding RNAs (sncRNAs) as part of their cargo, which can induce, modulate, and control a wide range of biological functions in target cells [2, 3]. It is believed that all eukaryotic cells release heterogeneous EVs to the extracellular space. Depending on their origin, EVs are called exosomes (when produced by endosomes or multivesicular bodies, MVB), or microvesicles (also known as microparticles), when produced and released from the cellular membrane [4]. Currently, no EVs recovery system allows their classification based on origin, partly due to the lack of specific surface markers. Because of this, the term “extracellular vesicles” is recommended when referring to these structures, regardless of the technique used to recover them [5, 6].

Normally, EVs can be found in interstitial tissues and body fluids like blood [7], saliva [8], urine [9], breast milk, and others [10]. Depending on the type and cell state, the cargo of EVs, which includes lipids, proteins, and nucleic acids varies, making them a fundamental part of the secretome that cells (whether close or distant) use to exchange information, influencing the response of target cells [11] and affecting various processes such as cell differentiation, proliferation, and immune response [12]. A significant aspect of the success of EVs as regulatory elements lies in their ability to encapsulate noncoding RNAs, both long and short [11]. Among the latter, small interfering RNA, microRNAs (miRs), YRNAs, piwi-interacting RNAs, and others, play crucial roles as key regulatory elements in gene expression [4]. Interestingly, the loading of these sncRNAs into the EVs is highly regulated, and some of them are exclusively loaded, suggesting specific functions for different groups of EVs [13].

EVs exhibit versatility, complexity, heterogeneity, and efficiency in both health and disease conditions. Importantly, due to their inconspicuous circulation within the immune system, EVs can transport pathogenic elements or their components [14]. For instance, during dengue virus (DENV) infection, EVs from saliva of infected mosquitoes have shown to increased viral spread [15]. Additionally, *in vitro* experiments demonstrated that DENV-infected mosquito cells produced EVs containing viral RNA and proteins capable of infecting both mosquito and mammalian cells [16]. A prior study in our laboratory revealed that U937 cells (a macrophages cell line) infected with DENV-2 produced EVs that when incubated on endothelial cells (EC) induced an increased in the transendothelial electrical resistance (TEER) and cell activation. This was associated with the overexpression of adhesion molecules like ICAM and the release of TNF-α, IP-10, IL-10, RANTES, and MCP-1, suggesting that these EVs triggered a defensive response in EC prior to viral exposure or infection [17].

DENV, belonging to the genus *flavivirus* within the family *Flaviviridae*, is the causative agent of the disease known as “dengue fever” recognized as the most important arbovirosis globally due to its elevated morbidity and mortality rates in both children and adults [18]. This disease is caused by any of the four DENV serotypes (DENV1-DENV4), transmitted through the bite of female mosquitoes of the *Aedes* genus [19, 20]. Clinically, DENV can cause a variety of symptoms, however, a subset of patients may experience complications involving vascular leakage associated to i) vasodilation, ii) monocytes, macrophages, and neutrophils recruitment to the affected zone and iii) the production of vasoactive agents [20, 21].

Endothelial dysfunction in the context of DENV infection may be attributed to the susceptibility of EC to the virus [22]. Nevertheless, both *in vitro*, or *in vivo* models as well as post-mortem tissue examinations indicate that the rate of EC infection tends to be low (2–5%) Consequently, tissue damage is associated with the impact of a plethora of soluble mediators released by immune cells and other cell types, including cytokines, chemokines, complement system factors, antibodies [21] viral proteins [23, 24], and others. The EC response to these agents is diverse and can encompass cell activation, alterations in barrier function, and even apoptosis [25]. EC activation involves the secretion of soluble mediators facilitating the adhesion of immune cells to the endothelial tissue [26]. Depending on the intensity of the EC response, it may allow controlled transmigration of immune cells and the passthrough of molecules (ions and proteins) to the cell stroma aiming to control and resolve the infection. If the balance between pro and anti-inflammatory factors is not restored, the response of all cells intensifies, leading to further damage to the endothelial tissue, enabling the entry of immune cells, molecules, and viruses into the protected endothelial tissues [21].

It is possible that EVs produced by immune cells and the EC themselves in response to infection play a significant role during DENV infection. However, the specific nature of the EC response to DENV infection, particularly concerning the production and cargo of EVs, as well as their effects on non-infected EC (NIC) is still unknown. Therefore, the present study aimed to evaluate and characterize the EVs produced by the human EC line EA.hy 926 infected with DENV-2. Additionally, the study assessed the functional effects of these EVs and their impact on virus genome replication after polarized EC were exposed to them.

## Materials and methods

### Virus propagation in C6/36HT cells

For all the infection assays, the DENV-2 strain S3 provided by the Colombian National institute of Health (INS) was utilized [27]. The virus was propagated in C6/36HT mosquito cells (ATCC CRL-1660), which were maintained at 33°C in L15 medium (Biowest, L0300). This medium was supplemented with 10% fetal bovine serum (FBS), non-essential amino acids (MEM NEAA, Gibco 1140–050), Tryptose phosphate broth (Gibco 18050–039), and L-Glutamine 2 mM (Biowest, X0550). Once the cells reached 80% confluence, 100 μl of viral inoculum at a multiplicity of infection (MOI) of 0.1 was diluted in fresh culture medium with 2% FBS. This mixture was added to the cell monolayer and incubated for 8–10 days until a cytopathic effect was observed. Subsequently, the cells underwent three freeze-thawing cycles, were mechanically detached using a scraper, aliquoted and stored at -80°C until further use. Viral titration was performed on BHK-21 cells as previously described [28]

### Cell culture and infection with DENV-2

EA.hy 926 endothelial cells (CRL-2922 ATCC) were cultured in DMEM supplemented with 10% fetal bovine serum (FBS) and antibiotics (penicillin 100 U/ml and streptomycin 100 μg/ml (Biowest). Upon reaching 80% confluence, cells were detached using trypsin-EDTA 1X solution (Biowest) and seeded onto Poly-L-Lysine-treated round glass coverslips (1,5 x10^4^ cells), 96-well plates (6 x10^4^ cells/well), 12-well plates (1,5 x10^5^ cells/well), or T75 cm^2^ flasks (7,5 x10^6^ cells). After 16 hours post-seeding (hps), cells were infected with DENV-2 at a multiplicity of infection (MOI) of 1 for at least 12 h.

The infection was carried using DMEM with 2% FBS and antibiotics [29]; then, viral inoculum was removed, cells were washed with PBS 1X, and subsequent procedures were performed based on the upcoming experiments. For EVs collection, cells were incubated in BIO-MPM-1 (Biological Industries) defined medium supplemented with 2 mM of glutamax (Biowest) for 48 h. In the case of cells seeded on plates, fresh DMEM plus 5% FBS was added, and infected cells were kept for 0, 3, 6, 12 and 24 hours at 37°C and 5% CO_2_. After the designated times, cell culture medium was removed and stored at 4°C. Cells were fixed with 4% paraformaldehyde (PFA), collected for PCR or western blot (WB) assays, or processed for viability assays. For the time zero, cells were processed immediately after viral removal. BHK-21 cells (CCL-10^™^ ATCC) served as infection control, and non-infected C6/36-HT supernatants were used as mock condition.

### Cell viability assay and viral RNA detection

After 16 h of seeding on 96-well plates, EA.hy 926 cells were infected. Subsequently, at the specified time points, supernatants were removed and stored at 4°C. Simultaneously, infected cells (IC) and NIC were incubated at 37°C for 2 h with Resazurin (44 μM, Invitrogen), diluted in DMEM. Following incubation, fluorescence was measured using the Clario Star (BMG, Exc 494 nm / Em 517 nm) and analyzed using the software MARS 3.41; data is shown in relative fluorescence units (RFU). Furthermore, lactate dehydrogenase (LDH) detection was performed using the collected supernatants and the Roche-Sigma 11644793001 kit. Data analysis is expressed as the percentage of cytotoxicity normalized to controls, as described in the manufacturer’s instructions. As a positive damage control, cells were incubated with Triton X-100 (1%) for 20 minutes (before adding resazurin) or for 1 hour before collecting the supernatants for the LDH assay.

The cells seeded on coverslips were infected for a maximum time of 48 hours post-infection (hpi). At 12, 15, 18, 24, and 48 hpi cells were fixed with 4% PFA for DENV detection through indirect immunofluorescence (IFI) assays. In brief cells were permeabilized with 0.3% Triton X-100 and treated with 10% goat serum blocking solution. Subsequently, cells were incubated with the specific antibody targeting the E viral protein, as previously described [30] and analyzed using the fluorescence microscope Axioimager M2 and its software Zen 2012 (Zeiss). Cell counting was performed using the Fiji/ImageJ software. Manual quantification was performed using the cell counter tool, and the data was confirmed using the infection counter plugin for automated quantification of the infected cells [31]. The total number of infected cells present in three slides (8 fields per slide), of two independent experiments was used to calculate the percentage of cell infection.

Additionally, total RNA was extracted from cells using the RTP DNA/RNA virus kit (Stratec) following the manufacturer’s instructions. RT-PCR was performed using the Luna Probe One-Step RT-qPCR kit (NEB), 200 ng of template, and specific primers [32]. Conventional amplification was conducted using a T-100 thermocycler (BIORAD) and the products were run in a 2% agarose electrophoresis gel, visualized using the Geldoc^™^ XR+ imaging system (BIORAD). This set of experiments was performed in triplicate in three independent experiments.

### Extracellular vesicles isolation and quantification

**Fig 1:**
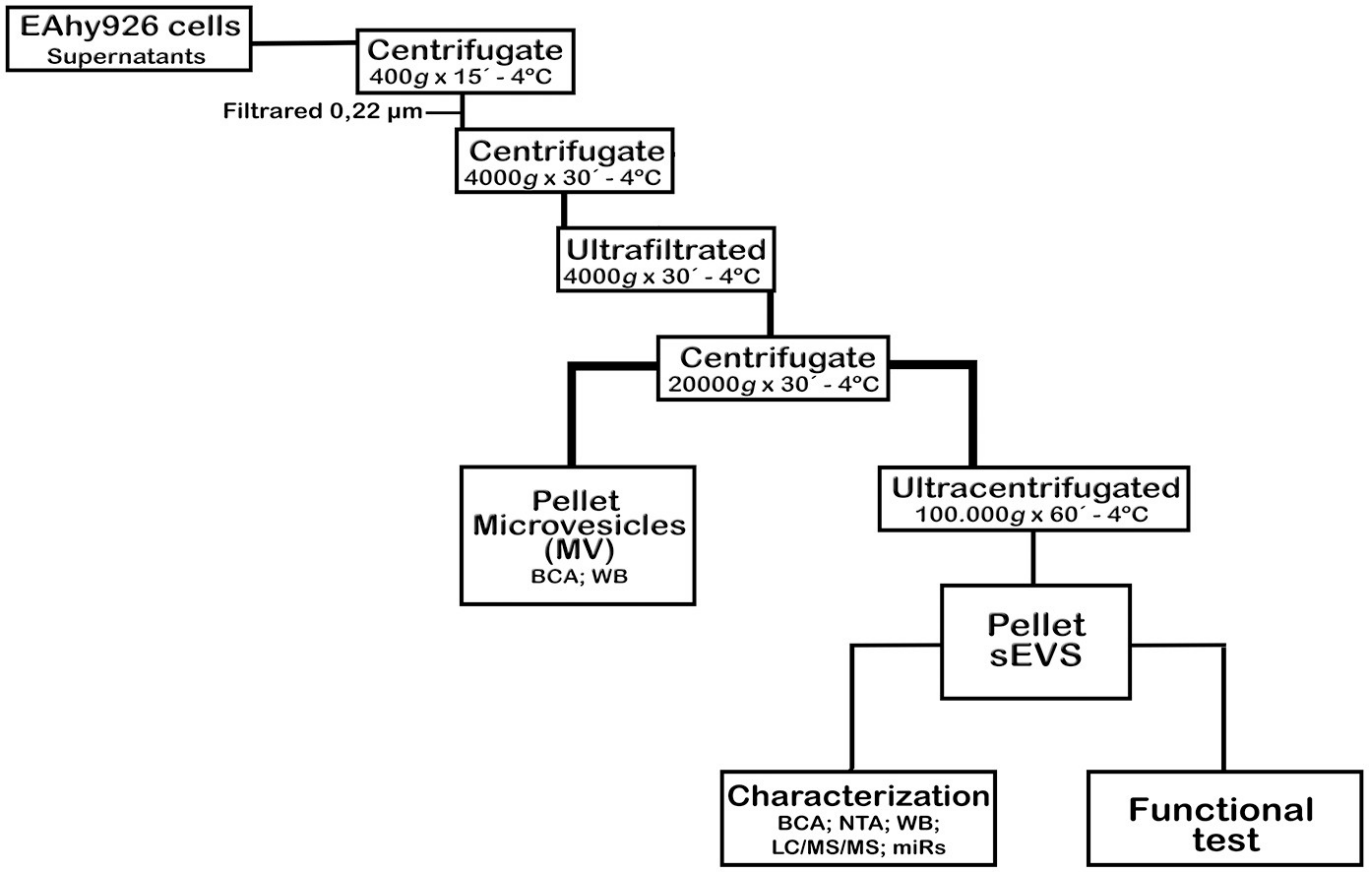
Workflow scheme for the isolation and processing of MVs and _s_EVs from cell supernatants.

Supernatants from IC or NIC seeded on T75 cm^2^ flasks (± 100 mL) were collected after 48 hpi and then centrifuged at 400 *xg* for 15 min at 4°C. Next, supernatants were passed through a 0,22 μM mesh and then centrifuged at 4.000 x *g* for 30 min at 4°C (rotor 1754 Hettich 380R) to eliminate bigger vesicles. Then, the remaining supernatants were ultra-filtrated using Amicon Ultra-15 MWCO 100K units, at 4.000 x *g* for 30 min at 4°C. For the isolation of microvesicles (MVs), the concentrated supernatants were centrifuged at 20.000 *xg* (rotor 1789L Hettich 380R) and the resulting pellets, containing MVs, were collected. Subsequently, the concentrated supernatants were ultracentrifuged (UC) at 100.000 *xg* for 60 min at 4°C (rotor TLS-55 factor K 100.2, Optima MAX-TL). The resulting pellets, referred to as small extracellular vesicles (_s_EVs) were resuspended in 100 μl of PBS 1X or 30 μl of Laemmli buffer 6X and stored at 80°C until use (Fig 1). To ensure the removal of any viral particles that might co-precipitate with the _s_EVs during ultracentrifugation (UC), an additional step was incorporated. The ultra-filtrated supernatants were treated with an acidic glycine solution (pH 3.0) for 30 minutes with constant agitation before the UC process. This treatment effectively inactivated any viruses present outside the extracellular vesicles.

For protein concentration determination, five microliters of vesicles pellets or whole cell lysates (WL) from EA.hy 926 NIC or IC were analyzed with the BCA technique, using a standard curve of bovine serum albumin (Thermo Fischer Scientific 23225) as reference. The resulting absorbance was measured using the Nanophotometer NP80 (IMPLEN).

### EVs characterization: Nanoparticle Tracking Analysis (NTA), and nano-scale liquid chromatography coupled with tandem mass spectrometry (LC/MS/MS)

For NTA analysis and Dynamic Light Scattering (DLS), non-centrifuged supernatants or _s_EVs from IC (_s_EVs-IC) or NIC (_s_EVs-NIC) were resuspended in 1 mL of PBS and analyzed using the NS300 Nanosight equipment and the Nanosight 3.4 Build 3.4.4 software, coupled to a CCD large sensor camera. Five different lectures were evaluated, and the mean or mode of particles/mL was reported (Cecoltec, Colombia).

For protein analysis using LC/MS/MS, 100 μg/μL of _s_EVs were resuspended in Laemmli buffer 6X and separated in a 10% SDS-PAGE gel at 200 volts during 5–10 min. Next, the gel was stained using a colloidal Coomassie solution, and the resulting band was cut and preserved in water. Proteins were identified and quantified using a Label-free approach and a nanoLC-MS/MS platform. Raw MS files were analyzed and searched against human and DENV databases using Maxquant (1.6.2.14). The parameters were set as follows: the protein modifications were carbamidomethylation (C) -fixed-, oxidation (M) -variable-; the enzyme specificity was set to trypsin; the maximum missed cleavages were set to 2; the precursor ion mass tolerance was set to 10 ppm, and MS/MS tolerance was 0.6 Da. Protein abundances were compared between _s_EVs-IC and _s_EVs-NIC, and log2 fold changes were obtained for each protein. Functional annotations for each protein were obtained from the Uniprot database (Uniprot Consortioum 2021) and manually corrected. Additionally, it was conducted an enrichment category analysis using the GOrilla tool [33]. Category terms for ranked protein lists in each condition were considered as enriched terms when their *p-value* ≤ 0.001 according to the minimum hypergeometric (mHG) statistical method.

### Small RNAs isolation, sequencing, and bioinformatic analyses

_s_EVs-IC or _s_EVs-NIC were treated with 100 μg/mL of RNase A (Thermo Fisher Scientific) and then UC at 100.000 × *g* for 1 h. Then, pellets were resuspended in the mirVana kit lysis buffer (Thermo Fisher Scientific), and phenol-chloroform solution was added, and the process continued according to the manufacturer’s protocol. Using the same kit, whole cell lysates from infected (WL-IC) and non-infected cells (WL-NIC) were processed. RNA concentration and quality were verified using the Bioanalyzer 2100 (Agilent Technologies). After that, libraries were prepared using the TruSeq^®^ SmallRNA Library Prep Kit (Illumina Inc.). Libraries were single end sequenced on one lane, for 51 cycles using the Hiseq2500 platform (Illumina Inc.). Raw fastq files were processed using the sRNAbench pipeline sRNAtoolbox [34]. The first step consisted of raw fastq files preprocessing, in which 3’ adaptor and barcode sequences were removed, then reads were filtered by size and length (15–41 nt). Clean reads were aligned to the human genome (GRCh38_p10_mp) and these were annotated using the hsa (Homo sapiens) small RNA annotations obtained from miRBase v22 database. sncRNA expression levels were estimated in Reads per Million (RPM). miRs expression files were compared to obtain the common and unique miRs expressed in _s_EVs and WL-IC and WL-NIC. Target genes were predicted using CyTargetLinker [35] app in Cytoscape v. 3.9. Human Target genes were retrieved from four databases: miRbase [36], TargetScan (TS) [37] and TransmiR [38]. The predicted target genes for unique expressed miRs were submitted to Gene Ontology (GO) enrichment analysis using the GOrilla tool [33]. GO terms for ranked gene lists and each condition was considered as enriched terms when their *p-value* ≤ 0.001 according to the minimum hypergeometric (mHG) statistical method. Additionally, the predicted target genes for unique miRs expressed in WL-IC were crossed with decreased proteins (using the gene protein ID) identified by mass spectrometry in _s_EVs-IC. Finally, miRs from _s_EVs were also analyzed for targets on the DENV-2 genome using the MirTAR database [39].

### Proteomics results validation: WB and proteasome assay

For WB assays, 20 μg/μL of protein from WL or the different pellets: microvesicles (MVs) and _s_EVs kept in Laemmli buffer 6X were run in 10–12% SDS-PAGE gels and transferred to a PVDF membrane. Then, membranes were blocked and incubated overnight at 4°C with specific antibodies to detect ALIX (1:1.000, CST #92880), CD63 (1:500, Abcam ab68418), TSG101 (1:5.000, Thermo MA1-23296), Histone 3 (H3, 1:2.000, CST #4499s), Calreticulin (CALR, 1:2.000 CST #4499s), and β-actin (1:10.000, Sigma 123M4876). Finally, membranes were incubated with the respective secondary HRP coupled antibodies (1:1000–1:10000, KPL 5220–0341; 5220–0336; 5220–0364). Results were observed using the SuperSignal^®^ West Pico Chemiluminescent substrate and the ChemiDoc^™^ Imaging System (BIO-RAD).

For proteasome assay, 3x10^6^ particles/ml of _s_EVs-NIC and _s_EVs-IC, resuspended in PBS, were incubated with the Suc-Leu-Leu-Val-Tyr-AMC fluorogenic substrate specific for calpains and 20S proteasome (ab142120). The samples were read every 30 min for at least 120 min, following the manufacturer’s instructions. The relative fluorescence emitted was measured at 360/460 nm and 25 flashes, using the TECAN Infinite 200 fluorometer. Data was recorded in RFU. Controls included WL-IC and WL-NIC along with _s_EVs pre-treated with Bortezomib 50 mM (B50) to inhibit of proteasome activity. This assay was conducted in 2 independent experiments with 2 replicates each.

### Evaluation of the functional effects of _s_EVs on EAhy926 endothelial cells

To assess the functional effects of _s_EVs on polarized endothelial cells, 6 x 10^4^ EA.hy 926 cells were seeded on the luminal side of semipermeable membranes of Corning Transwell inserts^®^ (0.33 cm^2^, 0.4 μm pore) pre-treated with collagen type IV and fibronectin (10 μg/ml). Once a TEER of 45 Ω/cm^2^ was reached, cells were exposed during 24 h to 3 million EVs from the different vesicle groups: I) _s_EVs-NIC, II) _s_EVs-IC, III) _s_EVs-IC^+4G2^ (the _s_EVs-IC treated with the neutralizing antibody 4G2 (1:10, Novus) for 1 h at 4°C), and IV) _s_EVs-IC^+4G2^_UV_ (an aliquot of _s_EVs-IC^+4G2^ was irradiated with UV light for 3h on ice), S1 Fig. Subsequently, the culture medium was removed, and the same cells were exposed to DENV-2 (MOI 1) for another 24h. TEER and Dextran Blue (DB) 2000 (2,4 mg/ml, Fine chemicals) permeability was assessed at different times as described in reference [17]. Finally, morphological changes of the cells were evaluated using the immunofluorescence technique with the markers phalloidin (CS 8940S) for actin structure evaluation and ICAM (SC 1511) for adhesion molecule visualization under the Axioimager M2 fluorescence microscope and the software Zen 2012 (Zeiss).

Additionally, total RNA was extracted from the EC seeded on transwell inserts^®^ using Trizol^®^ following the manufacturer’s instructions. The RNA was retro-transcribed and amplified using the Luna Probe One-Step RT-qPCR kit (NEB), and 200 ng of template. Specific primers were used to amplify the adhesion molecules VCAM and ICAM [17], the immune mediators IL-6, IL-8, MCP-1 and TNF-α and the transcripts for ALIX and TSG101, and normalizing data against β-actin. Data analysis was performed using the 2^ΔΔCt^ method [40]. For viral detection, absolute quantification was performed using a specific probe for E viral protein coupled to HEX fluorophore [41], and the results were compared to a standard curve that allowed quantification of viral copies/ml. A CFX96 thermal cycler (BIORAD) and its software Biorad-CFX Maestro 1.1 were employed to visualize the results.

### Statistical analysis

All experiments were conducted in triplicate and repeated in two or three independent experiments. The third experiment was performed only when the two first experiments had very different results. As controls, EA.hy 926 cells were treated with mock (supernatants of non-infected C6/36-HT mosquito cells) for infection and viability assays. Data normality was evaluated using the Shapiro-Wilk test (*p>0*.*05*). Viability assays were analyzed using ANOVA (*p<0*.*05*) and Bonferroni analysis as a post-hoc test. For LDH experiments, the Wilcoxon rank test (*p<0*.*05*) was performed. TEER and permeability assays were analyzed using ANOVA or Kruskall Wallis test and the Dunnet’s Multiple Comparison test (as post-hoc test) using as controls NIC or DENV-2 for each evaluated time point. Unpaired t-tests (*p<0*.*05*) were employed for specific comparisons. Finally, statistical significance for Infection percentage after _s_EVs treatment in the different groups was evaluated through Kruskal-Wallis and Mann-Whitney test.

## Results and discussion

### EA.hy 926 cells infection with DENV-2 did not affect cell survival

**Fig 2:**
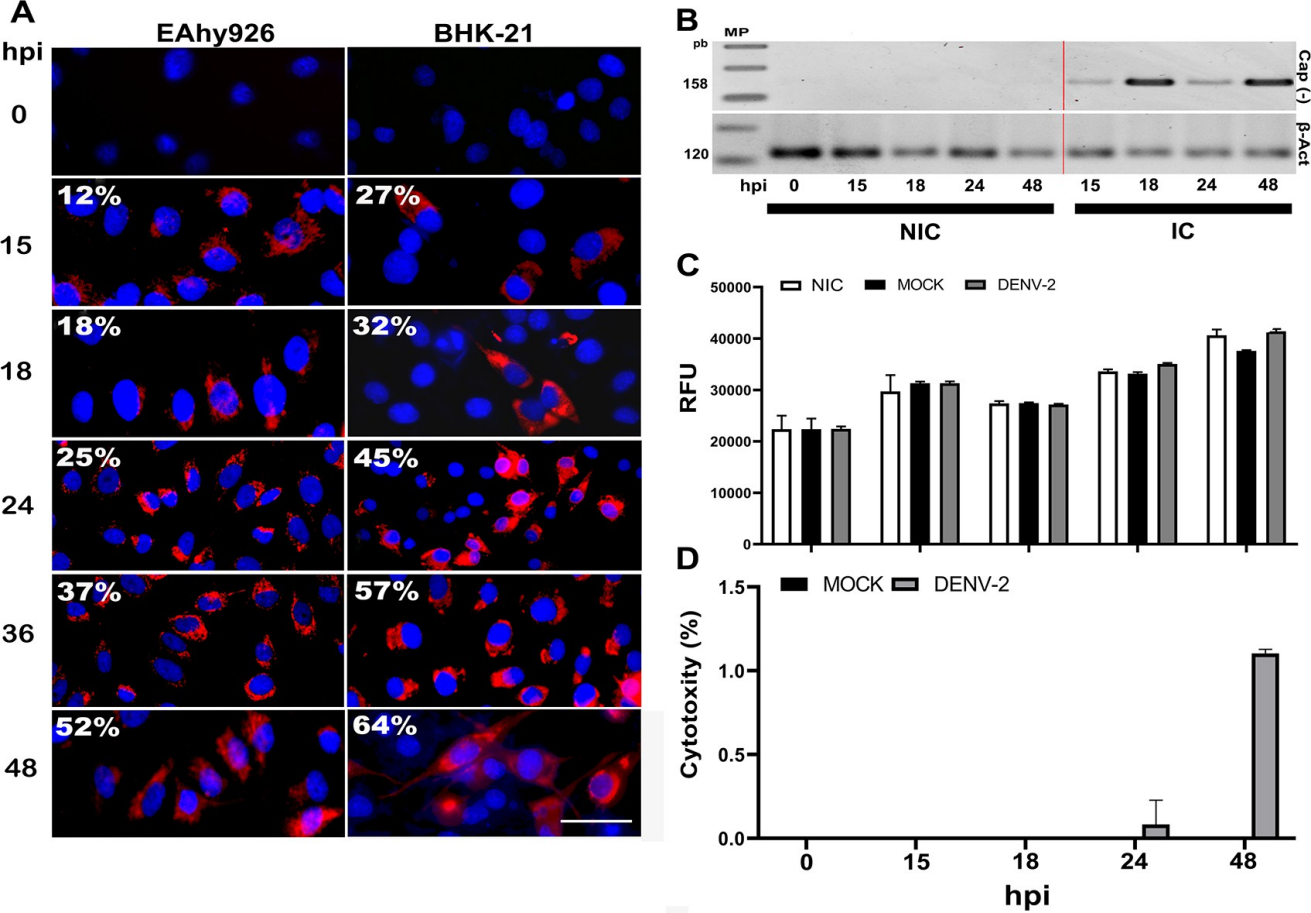
EA.hy 926 susceptibility to infection and cell viability assays. A) EC infection was confirmed by IFI for the viral “envelope, E” protein (red), while nuclei were stained in blue using DAPI. BHK-21 cells served as the infection control. The average percentage of the total number of infected cells is indicated in white for each culture at different hours post-infection (hpi). This calculation was performed by counting cells in eight fields in triplicate, in two independent experiments, using Fiji/ImageJ software. B) DENV-2 replication was verified by amplifying viral negative strands using the PCR technique and the forward primer for the capsid negative transcripts (cap -); β-actin was used as housekeeping control. C) Infection did not affect cell viability according to the resazurin and D) LDH assays. NIC: non-infected cells; IC: infected cells; mock: supernatants from non-infected C6/36-HT cells. All experiments were performed in triplicate in two independent experiments. *PCR original gels and cell counting raw data can be found on* https://doi.org/10.7910/DVN/BEKZOE, *Harvard Dataverse*.

Viral antigen was detected starting from 15 hpi, with a gradual increase in the number of infected cells observed over time. However, the number of infected EC was lower than observed for BHK-21 cells, which are known to be highly susceptible to DENV infection and in this case were used only as infection control. For instance, 45% of BHK cells were infected after 24h, while only 25% of EA.hy 926 cells were infected at the same time (Fig 2A). Viral replication in the EC was confirmed using the PCR technique, detecting negative viral strands in cell lysates (as viral replication signal), with a noticeable increase at 18 and 48 hpi (Fig 2B). Importantly, the infection did not affect cell survival during the evaluated times, as observed in the resazurin (Fig 2C) and LDH assays (Fig 2D). The latter showed only a 1.2% non-significant increase (*p>0*.*05*) in LDH release at 48 hpi (Fig 2D).

### EA.hy 926 infection induced a slight increase in the production of _s_EVs which mainly carried proteosome degradation associated proteins

At 48hpi we collected the EC supernatants without UC (nUC) to obtain the complete population of EVs including both large and small. These nUC EVs were characterized, and the sizes of the total vesicles were determined through DLS and NTA. The results indicated that NIC cultures produced small and medium vesicles of 22.6 nm and 262 nm respectively, with a mode of 304.3 nm and a concentration of 2,4x10^9^ particles/mL (S2A Fig). In contrast, IC cultures produced vesicles of 9.22 nm, 31.77 nm, and 248.7 nm with a mode of 188 nm and vesicle concentration of 3.1x10^9^ particles/mL. These findings confirm the heterogeneous concentration and sizes of EVs and demonstrate the effect of DENV-2 infection in the slight increase of produced EVs.

To classify the EVs, a centrifugation of 4000 *xg*, followed by another of 20.0000 *xg* was performed to eliminate larger EVs. A final UC of the ultrafiltrated supernatants was carried out to isolate _s_EVs (Fig 1). Given the physiological interest of the _s_EVs, these vesicles were also characterized. DLS results showed that _s_EVs-NIC had sizes of 8.13 nm and 46.56 nm, a mode of 222.6 nm, and a concentration of 2,2x10^9^ particles/mL. Meanwhile, _s_EVs-IC had sizes of 23.99 nm and 94.43 nm, a mode of 233.2 nm, and a concentration of 2,8x10^9^ particles/mL (S2B Fig). This indicates that DENV-2 infection induced a slight increase in size and _s_EVs concentration, with sizes closer to 100 nm compared to _s_EVs-NIC. The latter results showed that this EC line produced EVs of various sizes and confirmed that UC allowed the segregation of different EVs sets, keeping EVs with sizes ranged from 30–200 nm independently of the cells infection state.

**Fig 3:**
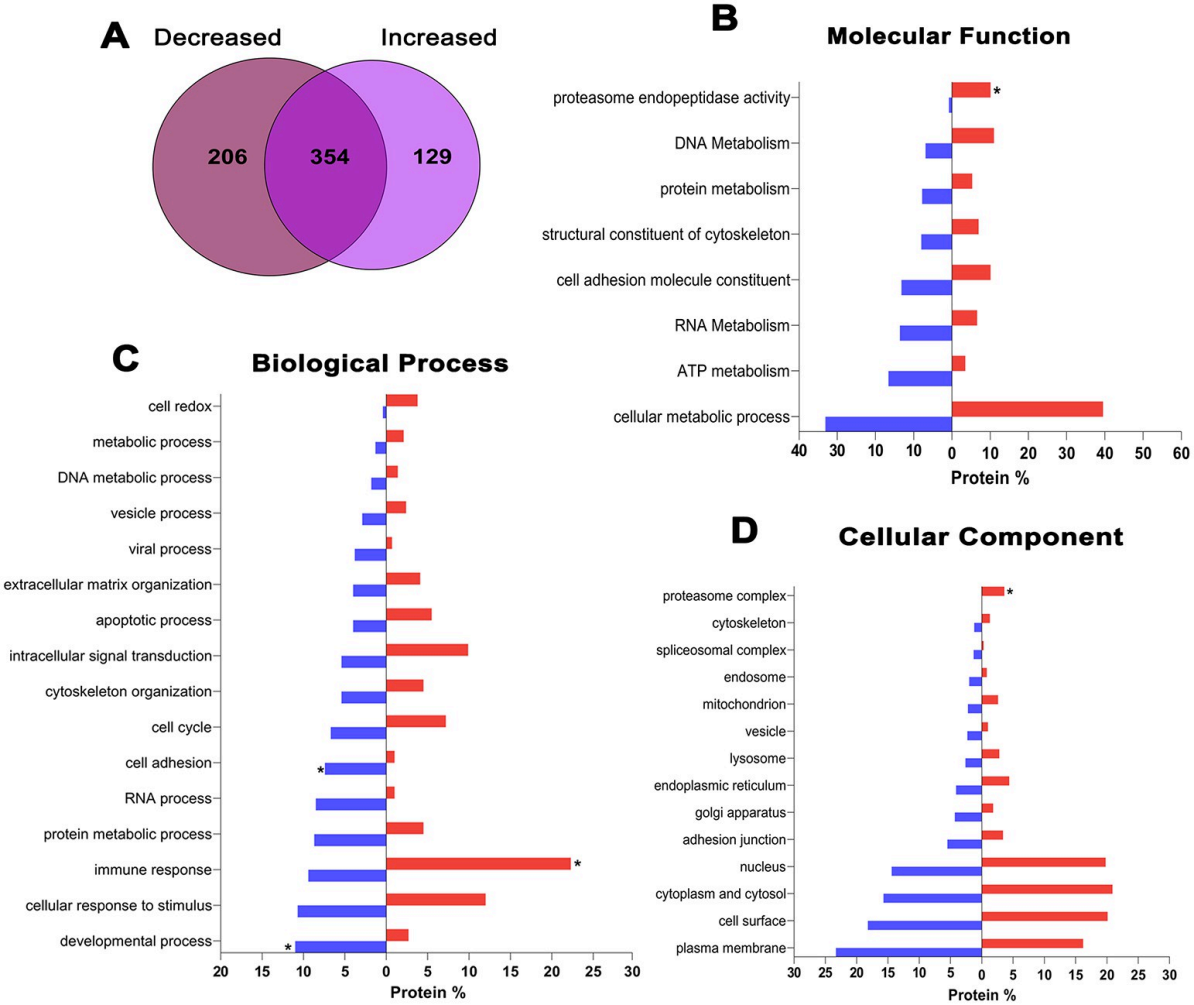
Proteomic analysis of _s_EVs. A) In comparison to _s_EVs-NIC, the results showed that _s_EVs-IC had 129 increased proteins and 206 were downregulated. B) The increased proteins mainly involved in molecular functions were related to proteasome activity. C) Regarding the biological processes, the significantly increased proteins were involved in immune response, while decreased proteins were mainly involved in cell adhesion and developmental processes. D) Consistent with the molecular functions, statistically significant proteins from cellular component were part of the proteasome complex, *p<0*.*01*.

The characterization of _s_EVs continued with mass spectrometry. The results revealed that _s_EVs-IC carried a total of 689 proteins, 129 of which were overexpressed, and 206 were downregulated compared to _s_EVs-NIC. 354 proteins showed no change in abundance in both conditions (Fig 3A). The specific definition of protein functions (GO terms) of _s_EVs-IC normalized to NIC, resulted in three groups: molecular function (Fig 3B), biological process (Fig 3C), and cell component (Fig 3D).

Most proteins exhibiting statistically significant differences among the groups were associated with the proteasomal complex and endopeptidase activity (increased), as well as cell adhesion and development (decreased) (Fig 3B–3D). Other GO terms with larger number of overexpressed proteins corresponded to immune and stimuli responses associated with signal translation, apoptotic processes, and redox activity. Meanwhile, the decreased proteins were mainly involved in processes related to development, stimuli response, and cell adhesion and migration (Fig 3B–3D, Table 1).

**Table 1:** GO analysis of the found proteins.

**GO enrichment Upregulated proteins**				
**GO level**	**Next common parental process**	**GO description**	**P-value**	**FDR q-value**
**BP**	cell surface receptor signaling pathway	interleukin-1-mediated signaling pathway	9,00E-07	3,49E-04
	Immune system and response	stimulatory C-type lectin receptor signaling pathway	9,00E-07	5,04E-04
		innate immune response activating cell surface receptor signaling pathway	9,00E-07	4,54E-04
		Fc-epsilon receptor signaling pathway	9,00E-07	2,84E-04
		T cell receptor signaling pathway	9,00E-07	2,52E-04
		antigen processing and presentation of exogenous peptide antigen via MHC class I, TAP-dependent	1,69E-06	4,26E-04
		antigen processing and presentation of exogenous peptide antigen via MHC class I	1,69E-06	4,05E-04
	Intracellular signal transduction	NIK/NF-kappaB signaling	9,00E-07	2,67E-04
	proteasomal protein catabolic process	proteasomal ubiquitin-independent protein catabolic process	7,21E-07	6,06E-04
		SCF-dependent proteasomal ubiquitin-dependent protein catabolic process	9,00E-07	3,24E-04
		anaphase-promoting complex-dependent catabolic process	9,00E-07	3,03E-04
	regulation of cell cycle	regulation of cell cycle G2/M phase transition	9,79E-08	4,93E-04
		negative regulation of cell cycle G2/M phase transition	3,40E-07	4,29E-04
		regulation of G2/M transition of mitotic cell cycle	6,62E-07	6,67E-04
		negative regulation of G2/M transition of mitotic cell cycle	9,00E-07	4,13E-04
	regulation of small molecule metabolic process	regulation of cellular amine metabolic process	1,27E-07	3,21E-04
		regulation of cellular amino acid metabolic process	3,40E-07	5,72E-04
		regulation of cellular ketone metabolic process	1,69E-06	4,48E-04
	regulation of transcription	regulation of DNA-templated transcription in response to stress	9,00E-07	6,48E-04
		regulation of transcription from RNA polymerase II promoter in response to stress	9,00E-07	5,67E-04
		regulation of transcription from RNA polymerase II promoter in response to hypoxia	9,00E-07	3,78E-04
**MF**	peptidase activity	threonine-type endopeptidase activity	1,27E-04	1,29E-01
		threonine-type peptidase activity	1,27E-04	6,46E-02
**CC**	proteasome complex	proteasome complex	1,69E-06	6,11E-04
		peptidase complex	1,69E-06	4,07E-04
**GO enrichment Downregulated proteins**				
**Gene Ontology**	**Next common parental process**	**GO Description**	**P-value**	**FDR q-value**
**BP**	Cell Motility	cell migration	3,37E-05	1,70E-01
	Cellular process	cell adhesion	8,76E-05	1,10E-01
	Biological process	developmental process	1,53E-04	1,54E-01
**MF**	Transcription regulatory activity	nucleic acid binding	1,21E-04	1,23E-01
**CC**	Cell periphery	plasma membrane	1,05E-06	3,78E-04
	Cell surface	cell surface	9,61E-04	1,74E-01
	Membrane bounded organelle	vesicle membrane	9,81E-04	1,42E-01

Increased and decreased proteins of _s_EVs-IC normalized to _s_EVs-NIC were grouped according to its biological process (BP), molecular function (MF) and cell component (CC). P-value and FDR q-value were calculated as mentioned in the methods section. FDR: False discovery rate.

Interestingly, 20S and 26S proteins, associated with proteasomal assembly and degradation were abundantly found, along with proteins of cell metabolic processes (Fig 3D). Following this, we performed a proteasome activity assay of the _s_EVs (NIC and IC) for at least 120 min. Results showed that the proteosome proteins loaded in the _s_EVs are active and as expected, proteasome proteins activity from the _s_EVs-IC (~5628,5 RFU average) were higher compared to _s_EVs-NIC (~4675 RFU average), S3A Fig.

The potential impact of these proteosome proteins on the metabolic activity and other processes of the target cells is a subject of interest [42, 43]. However, several aspects of the presence of proteasome 20S proteins in EVs remain unclear, including their role during DENV infection, their functionality upon being transferred to target cells, and their specific role in the EC infection model. Previous evidence has demonstrated the enrichment of these proteins in EVs from erythrocytes infected with *Plasmodium falciparum*, that when placed in contact with non-infected erythrocytes were functional and promoted the actin cytoskeleton rearrangement, altering membrane stiffness, allowing parasites invasion [44]. This effect highlights the pivotal role of EVs in infection, contributing to transmission processes with a more sophisticated strategy than previously reported. It is probable that the loading strategy of proteasome proteins into the EVs promotes viral replication (distal or local) making this a pro-viral mechanism, according to the evidence seen in the cytoplasm of SNB-19 and HEK293 cells, where the proteasome system is essential for ZIKV and DENV replication [45]. Additionally, it is conceivable that this strategy serves as a mechanism for metabolic control in infected cells, facilitating the disposal of misfolded or excessed proteins, like happens in tumor cells [46] or in lysosomal diseases [47].

Also, fifteen proteins were exclusively detected in _s_EVs-NIC, most of them involved in EVs biogenesis; proteins like MMP1 and MMP2, actin, actin binding proteins, tubulin, tubulin associated proteins, proteins involved in cell metabolic processes, transcription initiation factors and associated metabolic processes of nucleic acids, among others, were also found.

Invariant proteins were also detected in _s_EVs-NIC and in _s_EVs-IC such as: TSG101, MMP1 and MMP2, actin and actin-binding proteins, tubulin and associated proteins, transport-associated proteins, cellular metabolic processes, transcription initiation factors and proteins associated with metabolic processes of nucleic acids among others were also present. In particular, the RNA-binding protein Y-box-binding protein 1 (YBX1), which is key in the regulation of non-coding RNAs was found (S1 Table). To our knowledge, our results are the first to report the presence of the YBX1 protein in EVs from DENV infected EAhy.926 cells. YBX1 is a DNA and RNA binding protein, primarily detected in the nucleus and cell cytoplasm, with only a few reports of its presence in EVs [48]. In atherosclerosis, YBX1 suppresses lipid uptake by binding to CD36, promoting its mRNA degradation and reducing CD36 protein expression in macrophages. It also reduces the inflammatory response by regulating the NF-κB pathway [49], regulates MCP-1 mRNA stability in vascular smooth muscle cells (VSMCs) [50], and controls the switch between proliferation and differentiation in these cells by targeting GC box-dependent genes [51]. Some reports suggests that cytoplasmic YBX1 can influence viral assembly and release. Diosa-Toro et al., reported that YBX1 interacts with the viral genome and propose a model in which YBX1 enables the interaction between the viral nucleocapsid and the structural protein E of flavivirus, required for proper assembly of intracellular virus particles and their secretion [52]. However, it is unknown whether this protein loaded into EVs could exert the aforementioned functions. Therefore, it is essential to evaluate if the YBX1 present in EVs is bound to the YRNAs and what could potentially be their effects on the EC and other cells during the infection processes.

**Fig 4:**
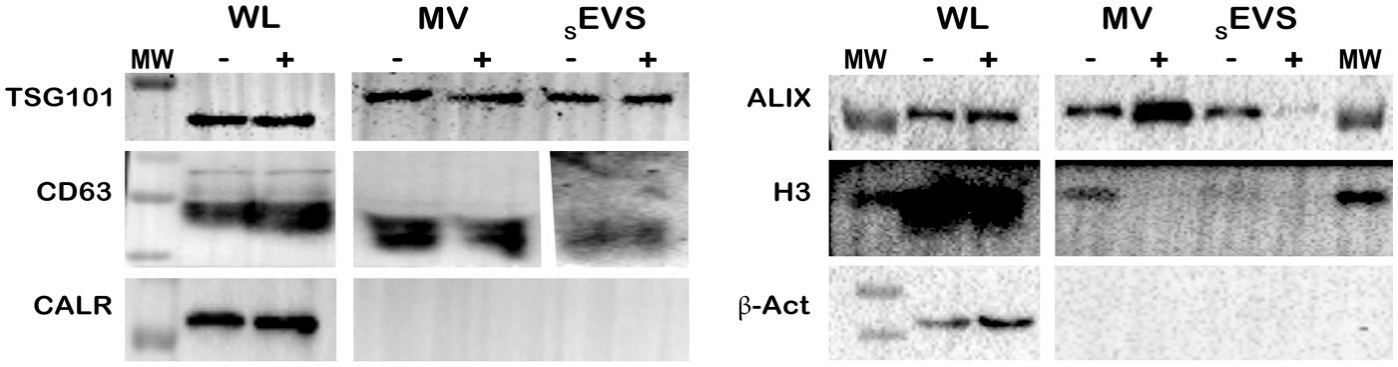
EVs characterization by WB. TSG101, Alix, CD63, Calreticulin (Calret), H3 and β-actin markers were evaluated in pellets of MVs and _s_EVs, compared to WL. The results showed that WL expressed all markers without significant differences between _s_EVs-NIC (-) and _s_EVs-IC (+). In contrast, the expression of Alix and H3 on MVs, and Alix only in _s_EVs exhibit changes in expression between EVs from infected (+) or non-infected (-) conditions. MW: molecular weight marker. *Raw data blots can be found on* https://doi.org/10.7910/DVN/BEKZOE, *Harvard Dataverse*.

Furthermore, we found proteins such as fumarate hydratase, dystroglycan 1, and O-fucosylpeptide 3-β-n-acetylglucosaminyltransferase exclusively in sEVs-IC. Other abundant proteins in these vesicles were Cytosolic purine 5’-nucleotidase (10-fold), Proteasome subunit β type-8 (PSMB8, 5-fold) and the Eukaryotic translation initiation factor 2 subunit 1 (EIF2S1, 4,4-fold) (S1 Table). On the other hand, downregulated _s_EVs-IC proteins were involved in functions like cell adhesion (PECAM, ITGAV, ITGAB1, ITGAB3), RNA and DNA metabolism (HNRNPA1, MCM3, VARS, DHX9, DNANB1 1, STAT1) among others. Interestingly, Alix (PDCD6IP) and the probable ATP-dependent RNA helicase (DDX5), were downregulated (S1 Table). The latter was also evidenced by WB results, which showed that Alix protein had a higher expression level in _s_EVs-NIC compared to _s_EVs-IC (Fig 4).

Furthermore, there was no difference between _s_EVs-NIC and _s_EVs-IC regarding CD63 and TSG101. H3 protein was absent in both sets of _s_EVs. Importantly, the WB assay confirmed that the different centrifugation steps for EVs isolation allowed the separation of different EVs sets, as evidenced by the different protein expression patterns observed in MVs. For example, MVs from IC (MVs+) exhibit higher expression of Alix (95 kDa) compared to MVs from NIC (MVs-) and the _s_EVs results. CD63 (55 kDa) and TSG101 (46 kDa) were similarly detected in MVs-IC and MVs-NIC, while H3 (17 kDa) protein was only found in MVs-NIC (Fig 4). MVs or _s_EVs were negative to β-actin (42 kDa) and Calreticulin (60 kDa) as depicted in Fig 4. Finally, a peptide of the NS5 viral protein was identified in the _s_EVs-IC, corresponding to the polymerase region located at its N-terminus (S3B Fig).

### _s_EVs-IC also carried small RNAs, including YRNAs and miR which were exclusive compared to WL

Small RNA sequencing resulted in four libraries (_s_EVs-IC; _s_EVs-NIC; WL-IC; WL-NIC), obtaining an average of 57.303.062 raw reads. Adapter trimming and barcode removal, 41.989.521 clean reads (on average) with sizes between 16 and 41 nt were obtained. From these cleaned reads, 26’131.472 were aligned to the human genome (GRCh38_p10_mp). These reads mapped to different classes of small RNAs, including YRNAs, mRNAs, and miRs (Table 2). Our results underscore the versatile sncRNA cargo carried by EVs. As previously reported, the loading of RNAs into EVs is a highly regulated, selective, and exclusive process [53].

**Table 2:** Small RNA sequencing data of _s_EVs (NIC and IC) and WL (NIC and IC).

	_s_EVs NIC	_s_EVs IC	WL NIC	WL IC	Mean
**Raw reads**	53’355.782	53’855.600	56’934.416	65’066.453	57’303.063
**Clean reads**	49’071.790	50’640.818	19’167.629	49’077.850	41’989.522
**Total Mapped reads**	24’881.501	24’033.597	15’704.402	39’906.389	26’131.472
**Unique Mapped reads**	168.808	285.311	266.462	698.197	354.695
**mRNA Read counts**	1’783.803	1’838.433	1’472.774	3’660.633	2’188.911
**yRNA Read counts**	79.438	75.258	2.189	13.554	42.610
**miRs Read counts**	7’038.109	4’838.135	37.084	97.281	3’002.652
**Other (rRNA, tRNA, snoRNA, snRNA) Read counts**	4’812.929	4’958.525	8’808.288	19’955.571	9’633.828
**Un-assigned Read counts**	11’167.222	12’323.246	5’384.067	16’179.350	11’263.471

Data compilation of raw reads, clean reads, and total and unique mapped reads. Data counts of the sncRNA found is also shown.

**Fig 5:**
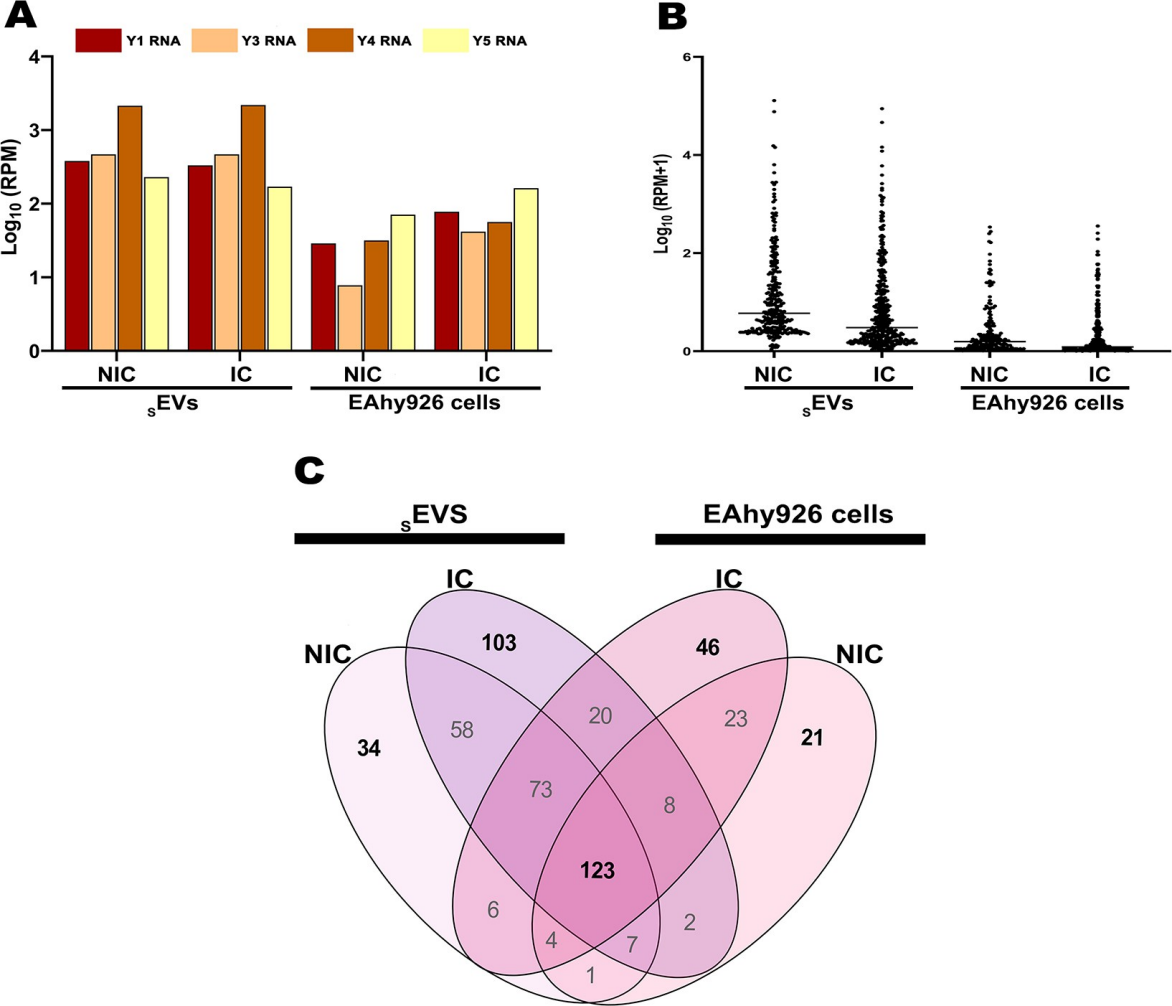
sncRNA found in _s_EVs and WL (NIC or IC). In general, (A) the _s_EVs had a larger load of sncRNAs like the 4 types of YRNAs and (B) miRs compared to WL (NIC or IC). (C) Unique miRs found for each evaluated condition.

Interestingly, all four types of YRNAs (Y1, Y3, Y4, Y5) were found in WL as in _s_EVs (IC and NIC). However, the expression levels comparison revealed that all four YRNAs were more abundant in _s_EVs-IC and _s_EVs-NIC, with YRNA4 having the highest number of reads in _s_EVs-IC corresponding to 2164,47 RPM. Infection induced an increase in the four YRNAs in WL-IC, especially for YRNA3 and YRNA5, with 41,62 y 163,98 RPM respectively (Fig 5A).

YRNAs are a class of regulatory sncRNAs, that are highly conserved in mammals and are expressed in all cells and tissue types, albeit with varying expression levels among them [53, 54]. The YRNAs are involved in the regulation of DNA replication, RNA stability and cellular stress responses, being key elements in pathological processes, like in atherosclerosis progression. To date, there are no reports suggesting the production and packing of this YRNAs in EVs produced by EC during DENV infection, nor is the function they might play during cellular response to DENV infection known.

Importantly, YRNAs have the capacity to produce (by themselves) miRs known as YsRNA. An example is the miR-1975, generated by the cleavage of YRNA5 mediated by caspases, which diversifies their regulatory capacity [55]. In cells infected with Influenza A virus (IAV), mir-1975 inhibits viral replication, and when loaded into EVs, it can induce the expression of interferon in recipient cells [56]. Similarly, during atherosclerosis, the YRNAs and some of its subproducts are essential for TNF-α secretion by macrophages [57]; they also regulate apoptosis and the Toll-like receptor (TLR)-dependent proinflammatory activity [58]. However, these hypotheses must be evaluated in our DENV infection model.

In addition, the recognition and loading of YRNAs into EVs as well as their functions, depend on their interaction with various proteins, including nucleolin, Ro60, La, hnRNPK, hnRNPI, ELAVL1 and YBX1 [53], the last one also loaded into the evaluated _s_EVs and discussed above.

Regardless of the infection condition, the miRs found in _s_EVs reached expression levels in the order of thousands of reads per million (RPM), whereas in WL miR expression levels were in the order of hundreds (Fig 5B, S2 Table). Of these, 123 were common to all conditions and 34, 103, 46 and 21 were exclusive by condition (hereafter referred to as unique), respectively (Fig 5C). However, no significant differences were found among the common miRs expression values of _s_EVs and WL-NIC or WL-IC; thus, the subsequent analyses focused on unique miRs of WL and _s_EVs (Fig 5C and S3 Table). The miRs with the highest expression levels in _s_EVs were hsa-miR-100-5p, hsa-miR-191-5p, hsa-miR-10b-5p, hsa-miR-21-5p, hsa-miR-99b-5p, hsa -miR-22-3p, hsa-miR-10a-5p and, hsa-miR-30a-5p (Table 3).

**Table 3:** List of the miRs highly expressed in the _s_EVs (IC and NIC) compared to WL (IC and NIC).

miR ID	Family	_s_EVs-NIC	_s_EVs-IC	WL-NIC	WL-IC		
**hsa-miR-100-5p**	MIR-10	129224	87945	164	256		
**hsa-miR-99b-5p**	MIR-10	76772	46169	24	92	**Expression value in RPM**	
**hsa-miR-10b-5p**	MIR-10	15473	12018	58	107		
**hsa-miR-191-5p**	MIR-191	14348	14493	249	92		>10K
**hsa-miR-21-5p**	MIR-21	6338	6031	340	193		>2K
**hsa-miR-22-3p**	MIR-22	4357	3911	1	59		>1K
**hsa-miR-151a-3p**	MIR-28	2795	2624	24	33		>500
**hsa-miR-10a-5p**	MIR-10	2759	2115	44	57		>100
**hsa-miR-501-3p**	MIR-362	2666	1362	0	2		>50
**hsa-miR-486-5p**	MIR-486	2508	1639	1	0		>30
**hsa-miR-30a-5p**	MIR-30	1994	1916	172	59		>20
**hsa-miR-92b-3p**	MIR-92	1612	1234	7	20		0–10
**hsa-miR-126-5p**	MIR-126	1408	1402	0	46		
**hsa-miR-221-3p**	MIR-221	1215	967	24	44		
**hsa-miR-181a-5p**	MIR-181	1104	1126	68	35		
**hsa-miR-423-5p**	MIR-423	822	696	7	3		
**hsa-miR-99a-5p**	MIR-10	660	457	0	2		
**hsa-miR-92a-3p**	MIR-25	653	689	46	31		
**hsa-miR-27b-3p**	MIR-27	638	581	0	33		
**hsa-miR-4485-3p**	N/A	173	130	276	356		

Data is given in reads per million (RPM).

### Unique miRs loaded in _s_EVs-IC or _s_EVs-NIC could regulate processes such as vesicle trafficking, gene expression, cell adhesion, and immune response in target cells

Target prediction resulted in 2732 and 6434 targets for the unique miRs enclosed in _s_EVs-NIC and _s_EVs-IC, and 2765 and 3264 for the unique miRs expressed in WL-NIC and WL-IC, respectively (S4 Table).

The predicted targets for unique miRs were subjected to a functional enrichment analysis resulting in 54 and 146 significantly enriched categories, respectively (S5 and S6 Tables).

To facilitate the analyses, these categories were manually grouped to the next common parental biological process, resulting in eight main processes, with cell adhesion, cell death processes and others, and immune response being the main targets regulated by those unique miRs loaded into _s_EVs-IC, while vesicular transport and processes related to gene expression (RNA transcription and translation) were the targets of the miR loaded exclusively in _s_EVs-NIC (Table 4). Highlighting that, the regulation of these targets by the miRs were exclusively found in one or the other experimental condition, meaning they were conditionally restricted.

**Table 4:** GO categories of the predicted targets of unique miRs from _s_EVs (NIC and IC) grouped by the main biological processes.

		Unique (%)	
Biological process	GO Description	sEVs-NIC	sEVs-IC
**Transport**	Vesicle trafficking	**26,4**	**15,9**
**Gene expression**	DNA templated transcription & translation	**11,3**	**0,0**
**Cell adhesion**	Cell—Cell adhesion & Cell substrate adhesion	**5,7**	**15,2**
**Cell death processes and others**	Apoptosis, cellular components organization or biogenesis.	**5,7**	**12,4**
**Immune system process**	Immune response	**0,0**	**10,3**
**Metabolic process**	Cellular metabolic process	**41,5**	**37,9**
**Cellular process**	Growth and Division	**7,5**	**7,6**
**Protein metabolic process**	Proteolysis	**1,9**	**0,7**

Data is shown as percentage of total targets for each biological process.

As previously reported in other infection models, there is a selective production and packaging of this RNAs with functions described in developmental, survival, metabolism and proinflammatory processes. This implies that this type of RNAs can modulate protein profiles and the cellular state of the target cell.

### *In silico* analysis of miRs from IC suggests an effect on the loaded _s_EVs proteins and on the regulation of DENV genome

**Fig 6:**
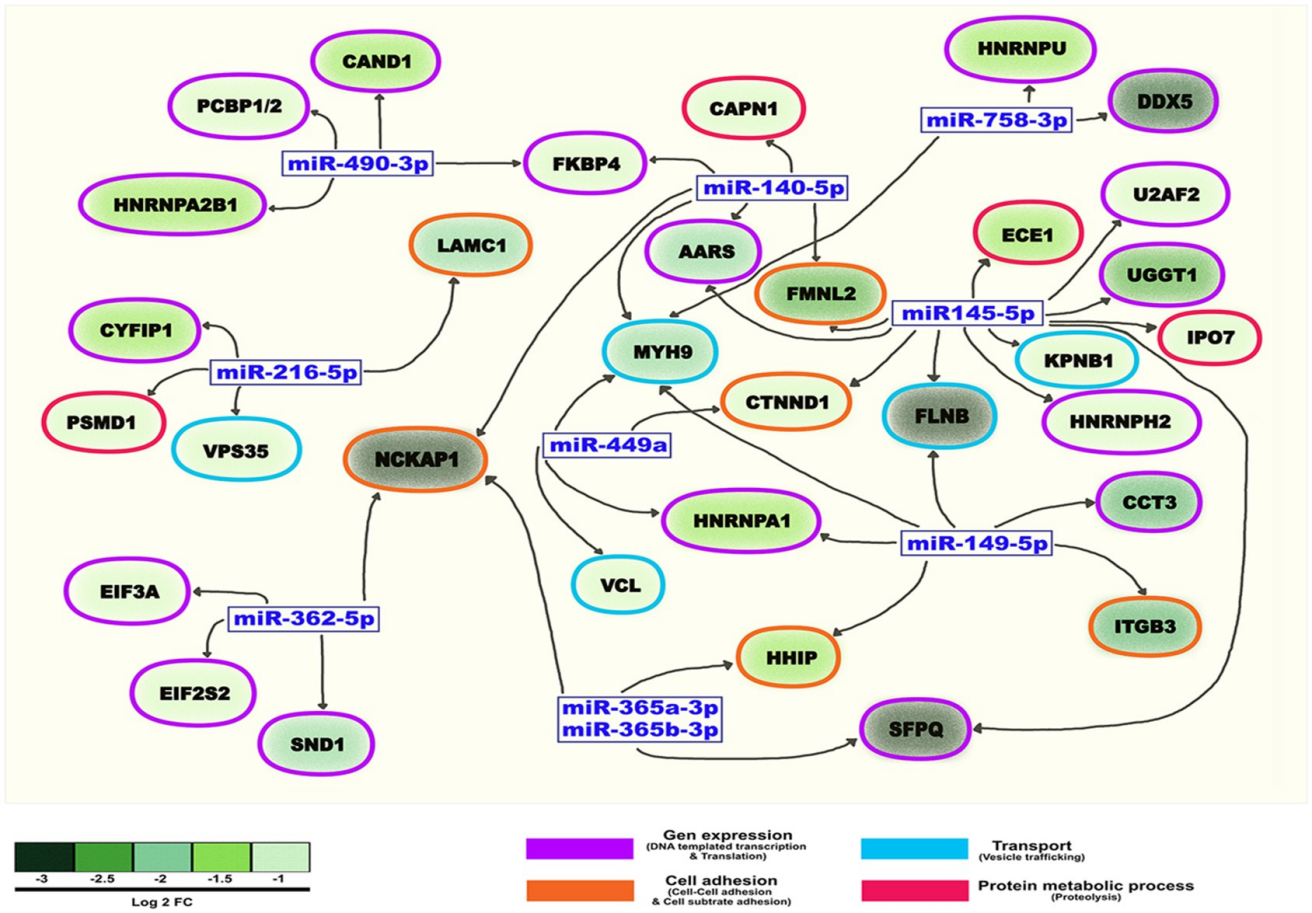
Network of the downregulated proteins present in _s_EVs-IC and the miRs from WL-IC that might regulate them. Compared to proteins from _s_EVs-NIC, thirty-three proteins present in _s_EVs-IC exhibited significant reduction, which might be attributed to the regulatory influence of 10 miRs identified in WL-IC. The oval shapes represent the proteins. Oval’s background green color indicates the decrease in protein regulation (FC: fold-change), and the edge color of the oval shapes represents the biological process associated with the respective proteins. miRs (blue) are part of a regulation network showed with black arrows.

Using an *in-silico* approach, we evaluated if the identified miRs present in cell lysates could explain the downregulation of some unique proteins found in the _s_EVs-IC. Specifically, 46 unique miRs from infected EC have the potential to downregulate 49 of the _s_EVs-IC proteins. Notably, 10 of these miRs could significantly impact the abundance of 33 of the proteins found in the _s_EVs-IC according to the Log2 FC (fold change) = < -1. These proteins were mainly involved in four biological processes: gene expression, cell adhesion, transport, and proteolysis. Some key miRs were the hsa-miR-140-5p and hsa-miR-145-5p, which could regulate the four above mentioned biological processes; hsa-miR-758-3p, hsa-miR-362-5p, hsa-miR-365a-3p, hsa-miR-365b-3p, that might be involved in the regulation of two of the mentioned processes, while cell migration and cell projection might be affected by three miRs: hsa-miR-216-5p, has-miR-365a-3p, has-miR-149-5p. Other proteins are regulated by one or two miRs (Fig 6).

Among the most downregulated proteins were FLNB, NCKAP1, SFQP, DDX5 followed by UGGT1, CCT3 and ITGB3. DDX5 is an RNA helicase involved in the regulation of RNA translation and transcription [59]. It also participates in the viral replication processes of SARS-CoV2 [60] and HIV [61], and modulates the RNA polymerase dependent on RNA like the NS5 protein of the Japanese encephalitis virus (JEV), a flavivirus like DENV [62]. The downregulation of these proteins loaded into the EVs might imply that target cells established an antiviral strategy in advance, decreasing the probability of viral replication when infected, or favor a proviral effect that controls the interferon response [63]. Cervantes-Salazar et al. reported that DDX5 interacts within the nucleus with the NS5 viral protein and induces its degradation after 48 hpi, resulting in a decrease in the production and signaling of interferon associated proteins, favoring viral replication. As mentioned before, NS5 seems to be an interesting target for viral regulation, and the EVs cargo of the EC (infected or not) might positively or negatively influence its activity as RNA polymerase depending on RNA. Additionally, we found a peptide of this protein in the _s_EVs-IC, confirming once again that EVs are key elements for the transport of viral components (proteins, peptides, genomic sections, etc.), which might influence target cells or contribute to a clearance strategy of the cells to eliminate excess or misfolded proteins [63].

Additionally, the *in-silico* evaluation revealed a potential regulatory interaction between the miR hsa-miR-216a-5p and the Alix mRNA, leading to a modest reduction in protein abundance (-0.96 Log_2_ FC). This finding aligns with the LC/MS/MS and WB results obtained for Alix, suggesting a plausible mechanism underlying the observed reduction in Alix protein levels. In addition, we conducted RT-qPCR assays to assess the production of Alix and TSG-101 transcripts in cells pre-treated with the different sets of _s_EVs and subsequently infected. The results revealed that TSG-101 transcripts remained relatively constant regardless of the cell pre-treatment, whereas Alix transcripts exhibited an increase when cells were pre-treated with _s_EVs-IC and its variants before infection (S4 Fig). This suggests that the regulation of Alix occurs after mRNA production and before protein translation, supporting our hypothesis of Alix protein regulation exerted by miRs such as hsa-miR-216a-5p. However, further experiments are required to validate this.

Also *in silico*, we evaluated the potential interactions between miRs present in _s_EVs-NIC and _s_EVs-IC, with the DENV-2 genome (NC_001474.2). Despite the low expression levels of these unique miRs, several of them were predicted to target specific regions of the viral genome. For instance, in _s_EVs-IC the hsa-miR-1908-3p (2.2 RPM) could potentially regulate the E viral protein-coding gene.

**Fig 7:**
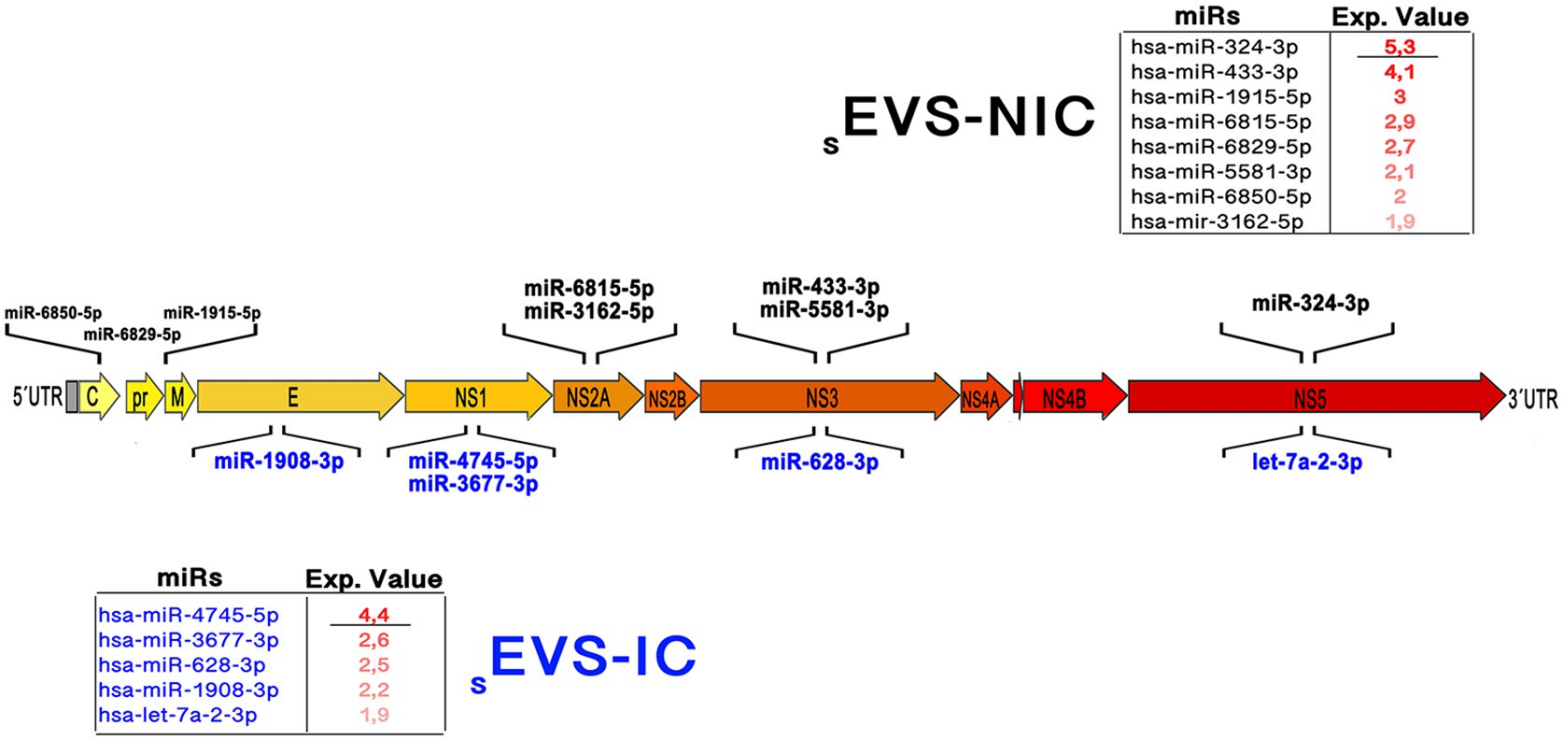
*In silico* analysis of miRs found in _s_EVs (NIC and IC) that might regulate the DENV genome. Some of the unique miRs from _s_EVs-NIC (black) and _s_EVs-IC (blue) that have as part of their targets, different regions of the viral genome. Exp value: expression value in RPM.

Additionally, hsa-miR-4745-5p and hsa-miR-3677-3p were predicted to target the NS1 gene. This suggests that this miRs might have an antiviral effect, given that the expression reduction of these proteins favors the complement activation, the lysis of the infected cells and the activation of IFN pathways associated to ISG genes [64]. On the other hand, hsa-miR-628-3p could regulate NS3, while let-7a-2-3p (part of the miR Let 7 family) might affect NS5 (Fig 7), suggesting an antiviral effect on DENV exert by these miR, similar to its reported effect on hepatitis C virus (HCV, another flavivirus), where it reduced the production of the RNA polymerase dependent of RNA, that is essential for HCV replication and other protein functions [65]. Nevertheless, this has to be validated in our model.

Interestingly, miRs exclusively present in the _s_EVs-NIC such as hsa-miR-324-3p, hsa-miR-433-3p, and hsa-miR-1915-5p, were also predicted to target the viral genes encoding NS5, NS3 and, M protein, respectively (Fig 7). These findings suggest a potential role for _s_EVs in modulating viral genome, and the differential regulation observed could be influenced by the cellular state of the EV-producing cells. This suggests that, constitutively, these sncRNAs packed into the EVs might serve as a defensive function against DENV. The packaging of these miRs into _s_EVs-NIC could offer a plausible explanation for the typically low DENV infection percentage reported in EC (no higher than 10%), and the observed reduction in viral genome replication in our model. However, all of these findings require further confirmation through additional studies to assess the negative regulation of these miRs in DENV replication, both in EC and other cell types.

This selectively loaded cargo of proteins and sncRNA allowed us to evaluate various aspects of endothelial physiology by exposing the EC cells to the _s_EVs and then to the viral stimuli, ultimately assessing cell activation, cell structural changes and viral genome replication.

### _s_EVs-IC pre-treatment activate the EC-NIC, maintained barrier integrity and reduced genome viral replication

It is known that EVs derived of some cell types can induce various effects on other cells, either be harmful or beneficial. For instance, EVs from leukocytes, erythrocytes, platelets, and hepatocytes, have been shown to influence adipose tissue and its metabolism [43]. Other studies have revealed that hepatic EVs are metabolically active and implicated in oxidative stress, endothelial dysfunction, and drug-induced liver injury [66–68].

To evaluate the effect of EVs in our DENV infection model in EC, the experimental design included the incubation of _s_EVs-IC with a neutralizing antibody for DENV clone 4G2 (_s_EVs-IC^+4G2^) and exposure of a portion of these _s_EVs to UV-light (_s_EVs-IC+^4G2UV^) to neutralize and inactivate any remaining viral molecules in the _s_EVs-IC (S1 Fig). After this, polarized non-infected EA.hy 926 cells were exposed to the different _s_EVs groups for 24 hours, followed by infection with DENV-2 (MOI 1) for an additional 24 hours, to evaluate morphological changes, barrier function, cell activation, and cell susceptibility to infection. Additionally, non-exposed cells to _s_EVs or only infected cells were evaluated as controls.

Importantly, EC pre-exposure to _s_EVs-NIC or _s_EVs-IC and derivates (prior to infection), kept the monolayer intact, with intense labeling for β-actin in the cytoplasm and fiber preservation in most of the cells. Nevertheless, the _s_EVs-IC stimuli induced an activated state of the EC, evidenced by an intense dotted cytoplasmatic labeling for VCAM and ICAM, compared to the results obtained when cells were pre-treated with _s_EVs-NIC (S5 Fig). Additionally, ICAM expression was also elevated by the _s_EVs-IC^+4G2^ and _s_EVs-IC+^4G2UV^ pre-treatment, while VCAM expression remained similar to _s_EVs-NIC (S5 Fig).

**Fig 8:**
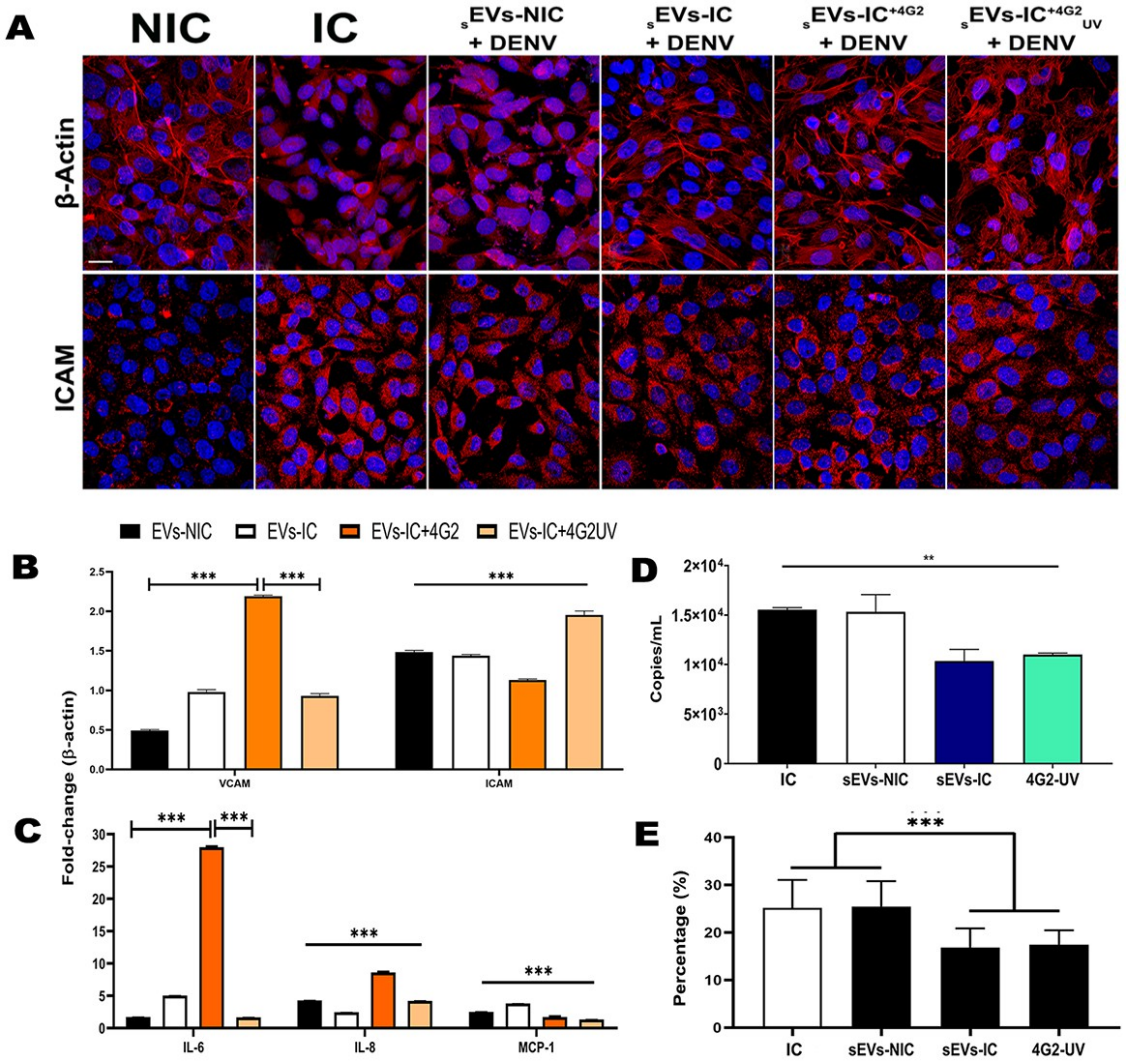
Functional assessment of _s_EVs on EAhy.926 polarized cells. A). EA.hy 926 cells were seeded on transwell inserts to allow cell polarization. Cells were exposed to the different _s_EVs (_s_EVs-NIC, _s_EVs-IC, _s_EVS-IC^+4G2^, _s_EVs-IC^+4G2^_UV_) during 24 h and then infected with DENV 2 (MOI 1) for another 24h. Monolayers were fixed, and then β-actin and ICAM were evaluated by IFI, Bar 50 μm. B) RT-qPCR was used for the detection of VCAM, ICAM, and C) IL-6, IL-8, TNF-α and MCP-1; data were normalized against β-actin and analyzed by the 2^ΔΔct^ method. D) Evaluation of EC susceptibility to infection after _s_EVs pretreatment by RT-qPCR. Monolayers pre-treated with _S_EVs-IC, or _S_EVs-IC^+4G2^_UV_, presented a statistically significant reduction of viral copies compared to non-exposed or pre-exposed cells to _S_EVs-NIC. E) Percentage of infection of EC when pre-treatment with _S_EVs. All experiments were evaluated in triplicate in 2 independent experiments. *Infected cell counts after pre-treatment with*_*s*_*EVs can be found on* https://doi.org/10.7910/DVN/BEKZOE, *Harvard Dataverse*.

After 24 hpi, in the IC controls (EA.hy 926 cells non-pre-treated with _s_EVs) infection induced morphological changes such as cell rounding and cell detachment associated to actin-fibers disassemble, evidenced by a dotted and scattered labeling in the cytoplasm. In contrast NIC maintained a typical morphology with continuous actin fibers throughout the cells (Fig 8A), although cells seem to be more separated from each other, and cell detachment was also evident. Assessment of cell activation, measured by the expression and distribution of ICAM revealed that in NIC controls (non-pretreated with _s_EVs), the protein was distributed smoothly through the whole cell cytoplasm with a few dotted aggregates. On the other hand, in IC controls, the protein was detected in the cytoplasm forming intensely marked and scattered aggregates, that was also seen when cells where pre-treated with _s_EVs-NIC and then infected (Fig 8A). However, EC pre-treatment with _s_EVs-IC and derivates (_S_EVs-IC^+4G2^ or _S_EVs-IC^+4G2^_UV_) helped to preserve the typical β-actin localization, with intense labeling in the cytoplasm and fiber structure preservation. These cells also showed an intense dotted cytoplasmic labeling of the adhesion molecule ICAM (Fig 8A). These results showed that in a similar manner _S_EVs-IC, _S_EVs-IC^+4G2^ or _S_EVs-IC^+4G2^_UV_ pre-treatment of the cells prevented damage to the EC monolayer integrity and maintained a similar cell activation status induced primarily by the viral infection (Fig 8A), which was also seen in the RT-qPCR assays (Fig 8B).

On the other hand, cell activation evaluated by the production of transcripts of soluble immune mediators like IL-6, IL-8, TNF-α and MCP-1 was also evaluated. Pre-treatment of polarized EC with _s_EVs-NIC did not cause significant changes in IL-6, IL-8, and MCP-1 after 24 hpi. However, it caught our attention that EC pre-treated with _S_EVs-IC^+4G2^ had a significant increase in IL-6 and IL-8 that might be associated to cell activation induced by the Fc receptors that interact with the 4G2 neutralizing antibody (Fig 8C). No TNF-α transcripts were detected under any of the tested conditions.

Additionally, we evaluated whether pretreatment of EC with _s_EVs modified DENV infection susceptibility. The RT-qPCR assay showed that monolayers pre-treated with _S_EVs-IC or _S_EVs-IC^+4G2^_UV_ before infection, presented a slight but statistically significant reduction of viral copies (10.346 and 10.993 copies/mL respectively), compared to non-exposed or pre-exposed cells to _S_EVs-NIC, which had 15.548 copies/mL of viral RNA (Fig 8D). Monolayers treated with _S_EVs-IC^+4G2^ showed the same reduction as _S_EVs-IC^+4G2^_UV_ (data not shown). Infected cells were also counted as mentioned in the methods section. Congruently, the results showed that EC pre-treated with _S_EVs-IC or _S_EVs-IC^+4G2^_UV_ were less infected (infection median of 17.3%) than cells pre-treated with _S_EVs-NIC which showed a similar percentage of infection to the IC control (infection median of 25%, Fig 8E), suggesting that _s_EVs-IC and derivates (_S_EVs-IC^+4G2^ and _S_EVs-IC^+4G2^_UV_) pre-treatment might limit viral replication (Fig 8D).

The changes induced by DENV in the actin cytoskeleton of the EC were also previously reported by our lab [17, 29]. Interestingly, when uninfected polarized EC cells were exposed to _s_EVs-IC and subsequently infected with DENV, this pre-treatment partially prevented the structural and functional damages while slightly reduced viral genome replication. Our findings suggest that compared to _s_EVs-NIC, _s_EVs-IC and derivates exert a protective effect at least during the first 24 hpi. This protective effect is manifested in the preservation of the actin cytoskeleton, reduction in DENV genome replication, and maintenance of EC activation, as evidenced by the expression of adhesion molecules like ICAM and VCAM, along with the production of immune mediators like IL-6 and MCP-1 (Fig 8). Notably, there was a decrease in IL-8 expression after infection in EC pre-treated with _s_EVs-IC; this cytokine has been reported to impact the endothelial barrier stability [69], providing partial support to our findings.

**Fig 9:**
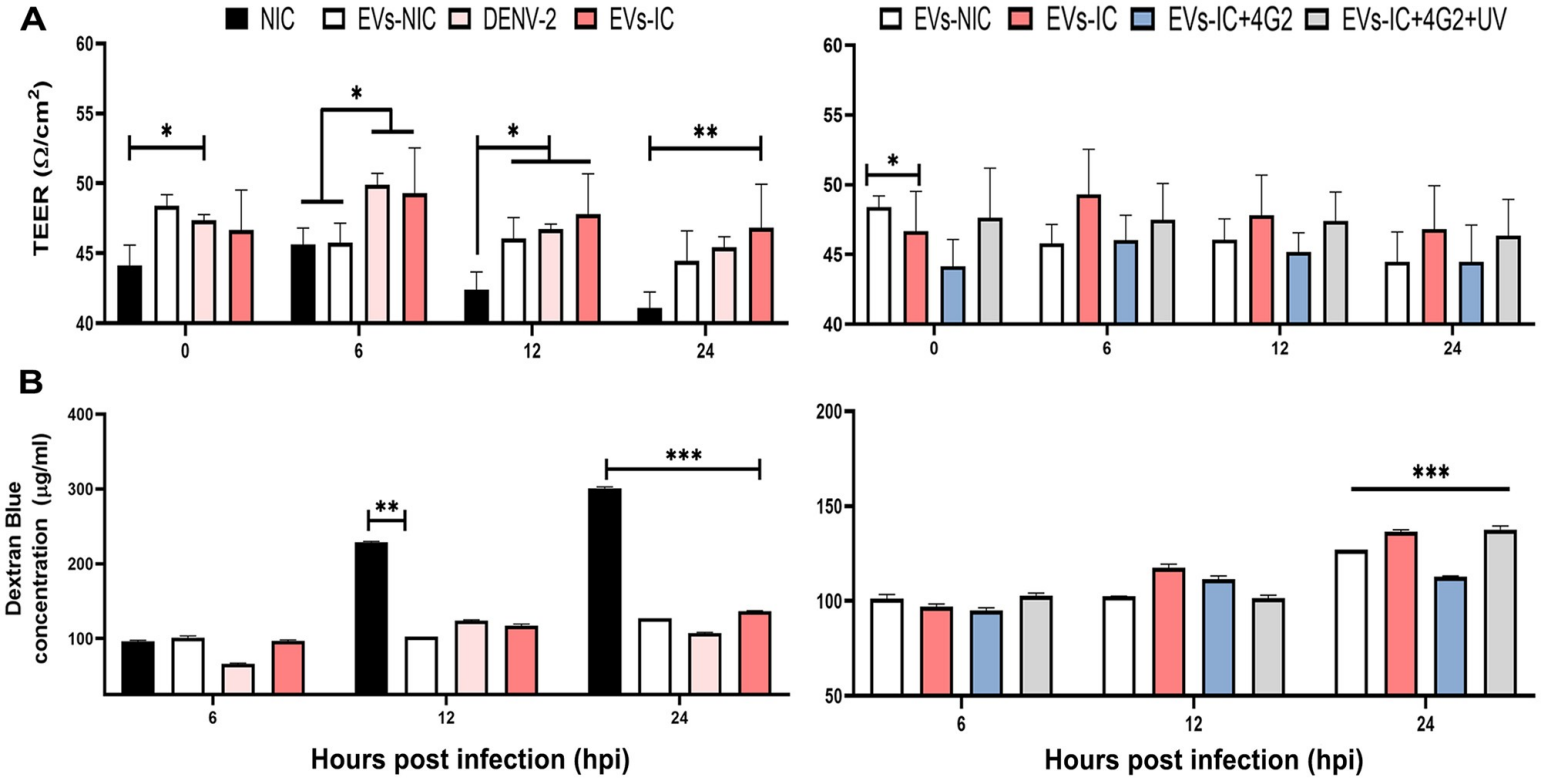
EC barrier function assessment. TEER (Ω/cm^2^) and DB permeability (μg/ml) results at different post-infection times after the polarized EA.hy 926 cells were pre-treatment with the _s_EVs. Results showed that pre-treatment with _s_EVs-IC activated the cells and preserved the monolayer integrity and cytoskeletal architecture compared to _s_EVs-NIC. These experiments were performed in triplicate, in 2 independent experiments.

Regarding monolayer stability and barrier function of the EC, we evaluated through TEER (Ω/cm^2^) and DB permeability. When the polarized EC reached a TEER of 45 Ω/cm^2^, cells were pre-treated with the different groups of _S_EVs during 24h, then supernatants were withdrawn, and cells were infected for another 24h to start TEER measurements and permeability evaluation at 0, 6, 12 and 24 hpi (Fig 9). In the NIC, TEER was stable at ~45 Ω/cm^2^ during the first 6 hpi and then diminished nearly to 40 Ω/cm^2^ at 12 and 24 hpi, which was related to DB permeability increase to 225 and 310 μg/ml, respectively, possibly related to cell renewal (Fig 9). In the case of IC control (non-exposed to _s_EVs), TEER values gradually increased to 50 Ω/cm^2^ during the first 6 hpi, related to the low permeability of the model with a minimum of 66 μg/ml of DB found in the lower chamber of the transwells. Interestingly, TEER reached 48 Ω/cm^2^ after pre-treatment with _s_EVs-IC and this increase remained stable during all experiment and was statistically significant when compared to _S_EVs-NIC. Similarly, when cells were pre-treated with the _S_EVs-IC^+4G2^ or _S_EVs-IC^+4G2^_UV_, TEER had a value of 48 Ω/cm^2^ and permeability also remained stable (Fig 9).

The previously described results suggest that pre-treatment of EC with _S_EVs-IC can induce an antiviral effect within the first 24 h post-infection, leading to a reduction in DENV replication. Our model showed a significant reduction (300-fold) in miR-21, which is known to modulate NS1 production [70]. We also observed an increase in several miRNAs associated with replication inhibition, including members of the let-7 family, miR-30, and miR-182-5p. In particular, miR-30 disrupts the NF-κB/IκBα negative feedback loop upon excessive NF-κB activation, leading to the upregulation of IFN-β and downstream ISGs, which ultimately inhibits DENV replication [71, 72]. Additionally, evidence elsewhere showed that miR-182-5p was found to decrease DENV-2 infection and replication in monocyte-derived macrophages (MDMs) [73].

However, miR-590, miR-105, and miR-34, which are involved in regulating tight junction-associated proteins or DENV-2-associated proteins, as well as miR-146b and miR-155, which modulate the immune response [74], were not detected.

Furthermore, we observed the activation of adhesion molecules like ICAM without significant changes in cell narrowing, allowing naïve ECs to maintain their barrier function. This effect may be attributed to the presence of proteins like actin and actin-binding proteins such as ARP2/3 and formin in the _s_EVs-IC. Proteomic analysis indicated that these proteins may explain the reduced structural damage observed in ECs pretreated with this group of _s_EVs before infection, thereby preserving monolayer stability and preventing a decrease in TEER. These proteins are not only involved in the biogenesis of EVs but also have the potential to regulate the actin cytoskeleton in target cells, promoting the stabilization of actin filaments and other actin-related dynamic processes, although the exact mechanisms remain unknown [75].

In general, various types of EVs, including exosomes and microvesicles, have been shown to induce EC activation and the production of soluble mediators, favoring an inflammatory environment and the endothelial dysfunction. However, studies by Jansen et al, suggests that the same EVs can exhibit anti-inflammatory, proliferative, and protective effects, reducing cell activation and preventing barrier damage [76]. This duality in effects implies that EVs may promote a mechanism of desensitization, preventing tissue damage when EVs interact with the same type of cells that produced them. This aligns with previous findings by our laboratory in a different DENV infection model, where EC exposed to EVs from infected macrophages demonstrated a similar protective effect [17].

## Conclusions

We would like to highlight three key elements of our infection model: I) The presence of YRNAs and miRs in both the _s_EVs from IC or NIC, which might explain the decrease in viral genome replication after _s_EVs pre-treatment of polarized cells. II) _s_EVs-IC and their cargo could prevent (at least during the early moments of infection) the structural changes of the EC and diminish cell infection aiming to keep their barrier function. III) A moderate expression of adhesion molecules seems to favor the barrier function stability, showing the EC resilience during the first DENV post-infection hours. Nevertheless, there are several aspects that need to be confirmed about the importance of _s_EVs during DENV infection in EC, like the role of sncRNAs, as well as the loaded proteins in processes like infection regulation, survival, and activation of infected and non-infected cells.

## Supporting information

S1 Fig Scheme of the method used to neutralize any possible viral particle present in the UC vesicles. (TIF)

S2 Fig NTA and DLS of A) non-UC supernatants or B) _s_EVs from NIC or IC. Results showed the heterogeneity of EVs sizes and the rise in its amount when cells were infected. (TIF)

S3 Fig Proteomic analysis complement. A) The possible activity of the proteasome complex proteins in the _s_EVs was assessed. Results showed that these proteins were active, where _s_EVs-IC proteasome proteins had a higher activity than the ones loaded into the _s_EVs-NIC. As comparing controls, the proteasome activity from WL (NIC and IC) was also analyzed, and pre-treatement of _s_EVs with B50 was used as proteasome proteins activity control. Results are representative from in two independent experiments with two replicates. B) Proteomic results showed the presence of a small peptide of the NS5 viral protein loaded in the _s_EVs-IC.
(TIF)

S4 Fig Transcript production of Alix and TSG101. RNA was extracted from cells pre-treated with the different sets of _s_EVs and subsequently infected. cDNA amplification for Alix and TSG101 revealed that TSG-101 transcripts remained relatively constant regardless of the cell pre-treatment, whereas Alix transcripts exhibited an increase when cells were pre-treated with _s_EVs-IC and its variants before infection.
(TIF)

S5 Fig Changes in EC after _s_EVs pre-treatment. Prior infection, no morphological changes were evident in the EC; actin filaments remained stable, and cells seemed polygonal or elongated. The monolayer was confluent; however, some spaces were especially evident in the monolayer treated with _s_EVs-IC, which might be due to cell renewal processes, without being possible to rule out if this is an effect of the _s_EVs. Pre-treatment of the EC with the _s_EVs-IC caused cell activation that was more evident when cells were marked with the ICAM antibody.
(TIF)

S1 Table List of proteins found in _s_EVs-NIC and _s_EVs-IC. (XLSX)

S2 Table List of miRs found in _s_EVs-NIC and _s_EVs-IC. (XLSX)

S3 Table List of unique miRs found in _s_EVs-NIC and _s_EVs-IC. (XLSX)

S4 Table List of targets of the unique miRs from _s_EVs-NIC and _s_EVs-IC. (XLSX)

S5 Table Predicted targets of the unique miRs from _s_EVs-NIC by BP. (XLSX)

S6 Table Predicted targets of the unique miRs from _s_EVs-IC by BP. (XLSX)

## Abbreviations

B50: Bortezomib 50 mM
BCA: bicinchoninic Acid
BP: biological process
Calret: calreticulin
CC: cell component
DB: dextran blue
DDX5: Probable ATP-dependent RNA helicase
DENV: dengue virus
DLS: Dynamic Light Scattering
EC: endothelial cells
EC-IC: infected endothelial cells
EC-NIC: non-infected endothelial cells
EIF2S1: eukaryotic translation initiation factor subunit 1
EVs: extracellular vesicles
FBS: fetal bovine serum
FDR: false discovery rate
Hpi: hours post-infection
Hps: hours post-seeded
IAV: influenza A virus
IC: infected endothelial cells
IFI: indirect immunofluorescence
IL-10: interleukin 10
IP-10: interferon gamma-induced protein 10
LC/MS/MS: Liquid chromatography–tandem mass spectrometry
LDH: lactate dehidrogenase
MCP-1: monocyte chemoattractant protein-1
MF: molecular functions
miRs: micro RNAs
MOI: multiplicity of infection
MVB: multivesicular bodies
MVs: microvesicles
MVs-IC: microvesicles from infected cells
MVs-NIC: microvesicles from non-infected cells
MW: molecular weight marker
N/A: not applicable
NIC: non-infected endothelial cells
NTA: Nanoparticle Tracking Analysis
PDCD6IP: Alix protein synonim
PFA: paraformaldehyde
PMSB8: proteasome subunit β type 8
RANTES: regulated on activation, normal T cell expressed and secreted
RFU: relative fluorescence units
RPM: reads per million
_s_EVs: small extracellular vesicles
_s_EVs-IC: small extracellular vesicles from infected cells
_s_EVs-NIC: small extracellular vesicles from non-infected cells
sncRNA: small non-coding RNA
TEER: transendothelial electric resistance
TNF-α: tumoral necrosis factor alpha
UC: ultracentrifugated
WB: western blot
WL: whole cell lysates
WL-IC: infected whole cell lysates
WL-NIC: non-infected whole cell lysates
