# Supplementary figures and images for: Small extracellular vesicles from the human endothelial cell line EA.hy 926 exert a self-cell activation and modulate DENV-2 genome replication and infection in naïve endothelial cells
Source: PLoS One. 2024 Sep 26;19(9):e0310735. doi: 10.1371/journal.pone.0310735 (PMC11426460; doi:10.1371/journal.pone.0310735)

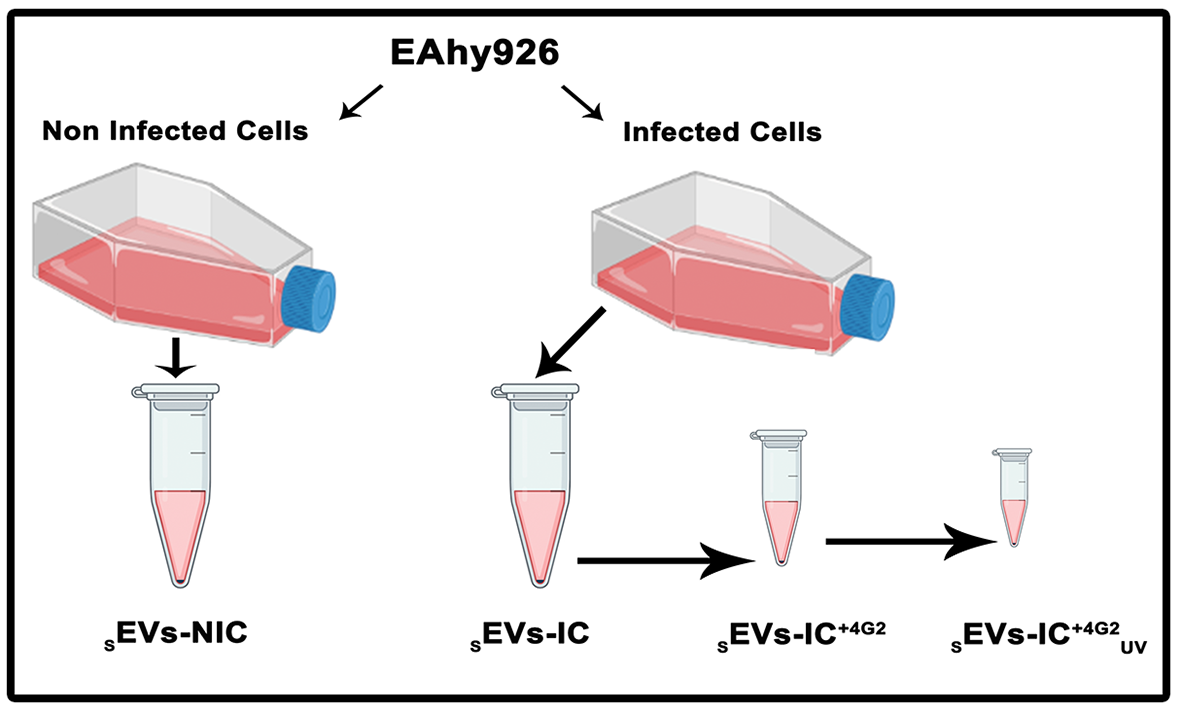

Supplement: S1 Fig — (TIF) [file pone.0310735.s001.tif]

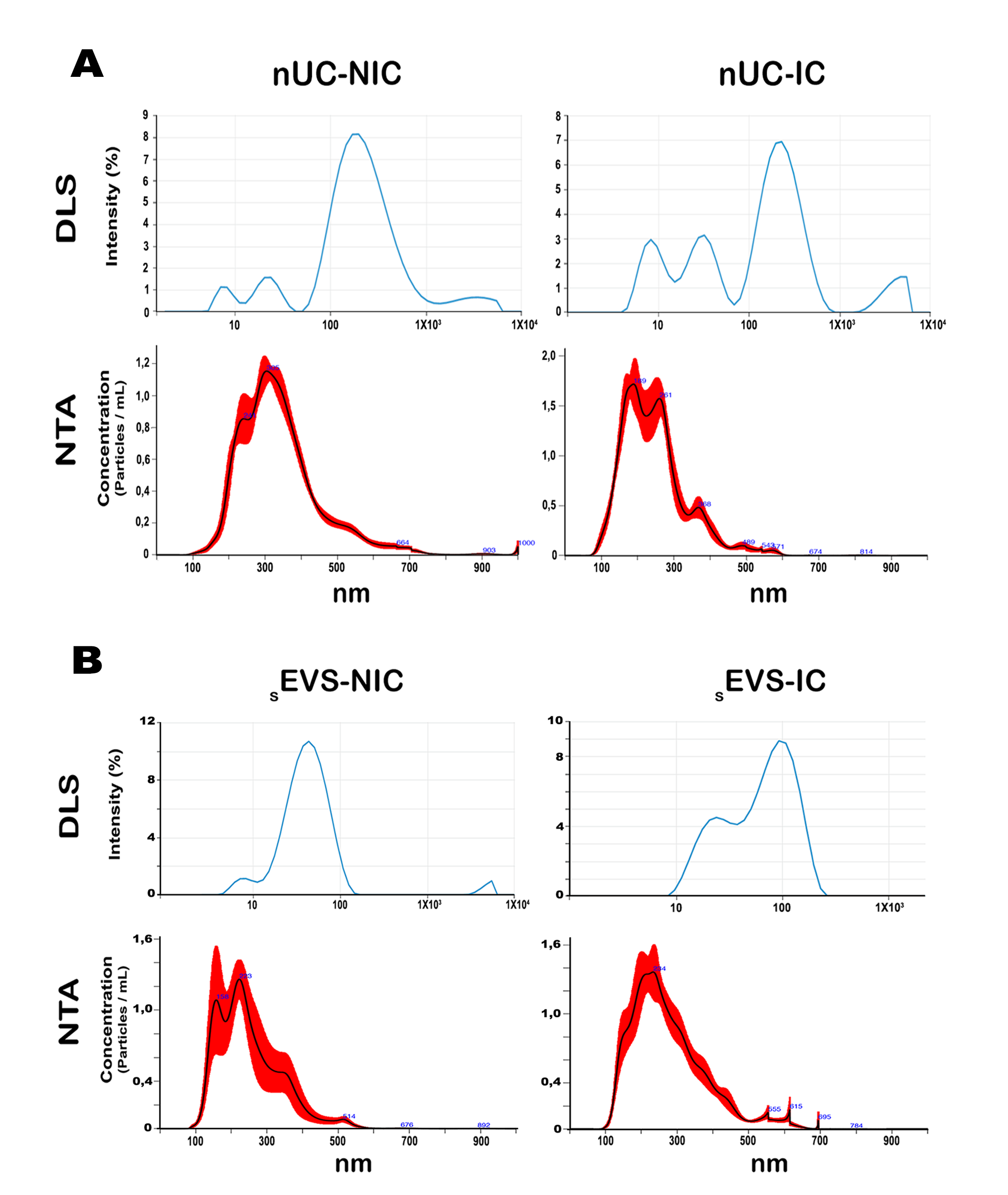

Supplement: S2 Fig — (TIF) [file pone.0310735.s002.tif]

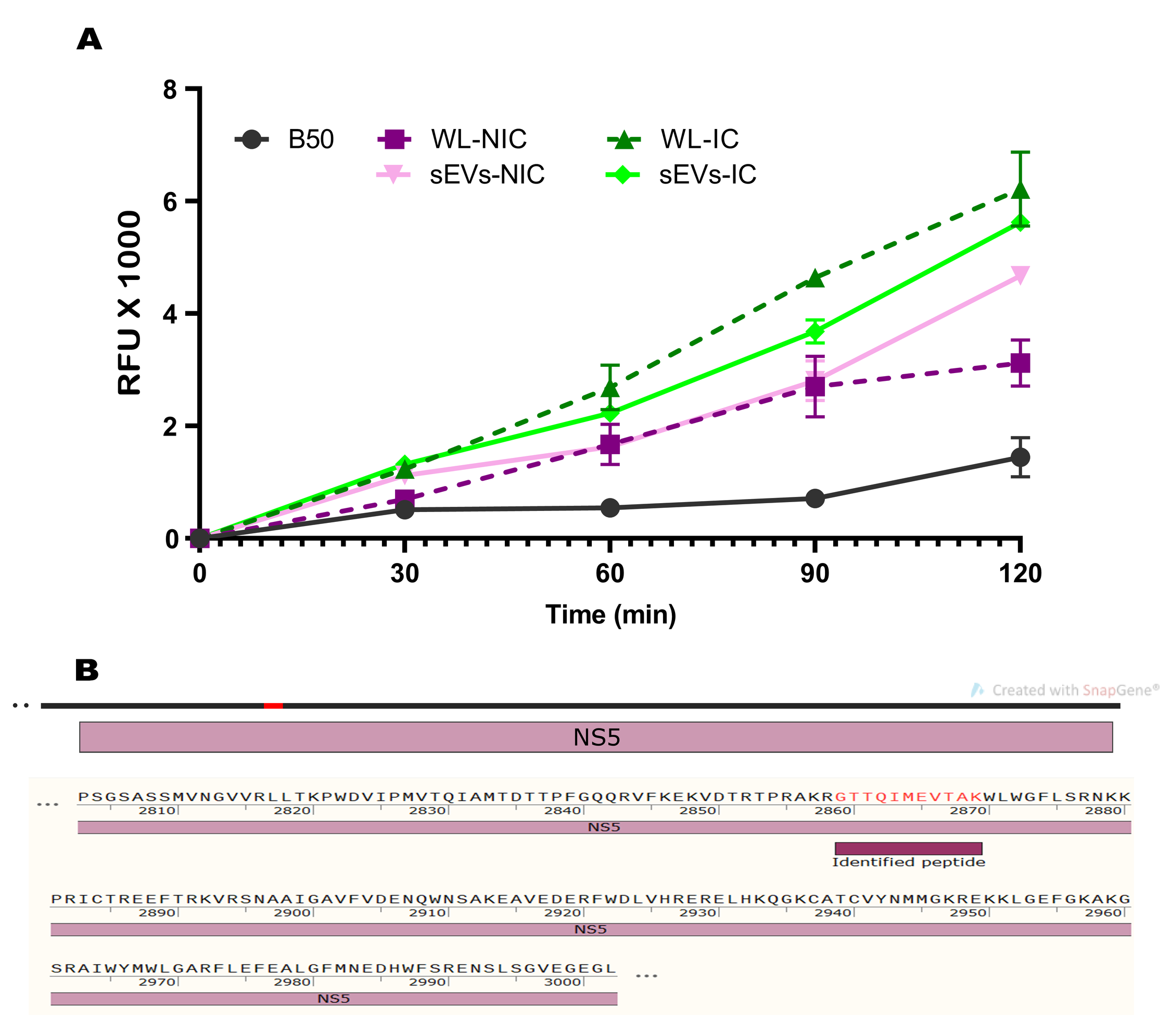

Supplement: S3 Fig — A) The possible activity of the proteasome complex proteins in the sEVs was assessed. Results showed that these proteins were active, where sEVs-IC proteasome proteins had a higher activity than the ones loaded into the sEVs-NIC. As comparing controls, the proteasome activity from WL (NIC and IC) was also analyzed, and pre-treatement of sEVs with B50 was used as proteasome proteins activity control. Results are representative from in two independent experiments with two replicates. B) Proteomic results showed the presence of a small peptide of the NS5 viral protein loaded in the sEVs-IC. (TIF) [file pone.0310735.s003.tif]

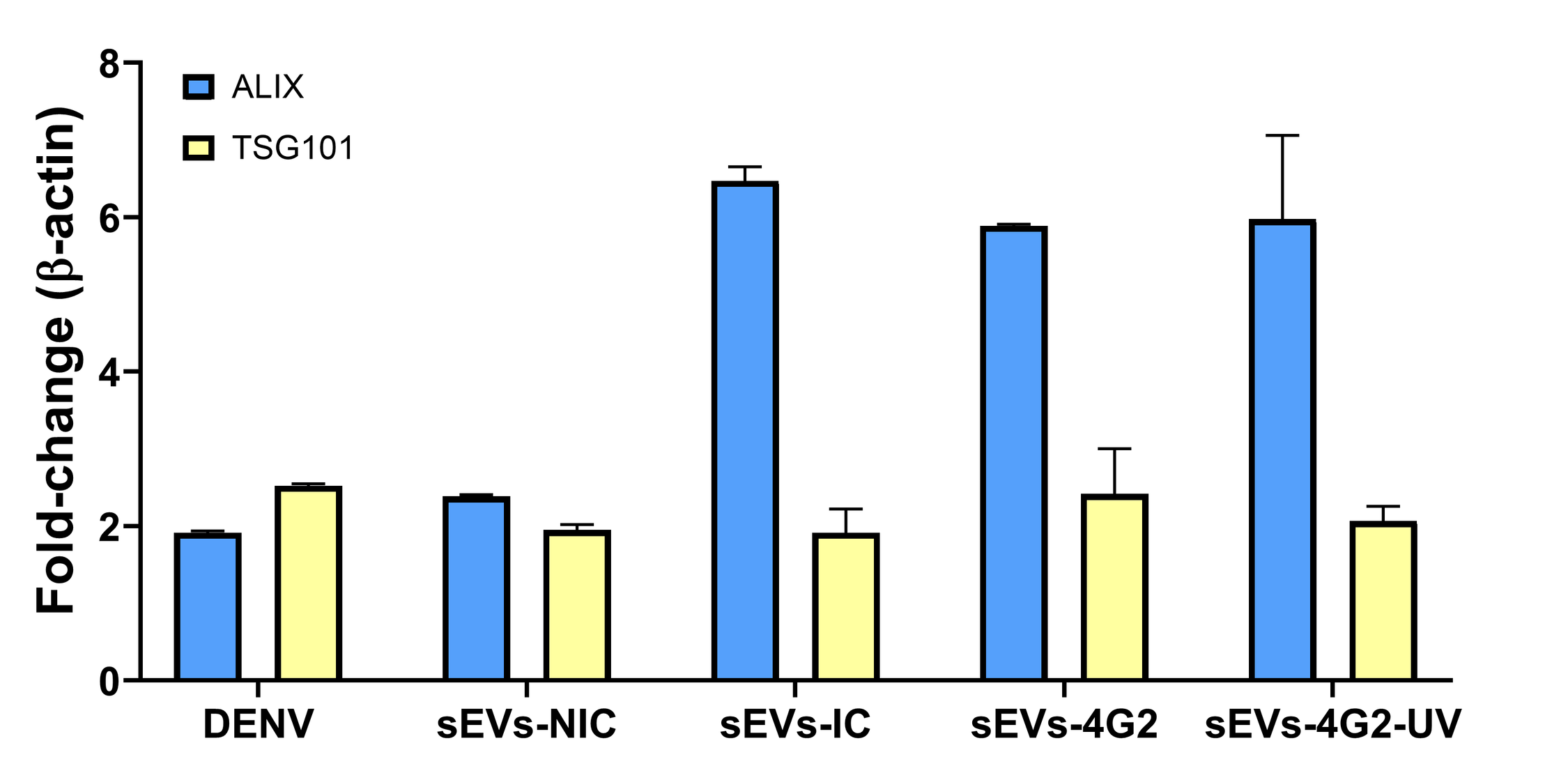

Supplement: S4 Fig — RNA was extracted from cells pre-treated with the different sets of sEVs and subsequently infected. cDNA amplification for Alix and TSG101 revealed that TSG-101 transcripts remained relatively constant regardless of the cell pre-treatment, whereas Alix transcripts exhibited an increase when cells were pre-treated with sEVs-IC and its variants before infection. (TIF) [file pone.0310735.s004.tif]

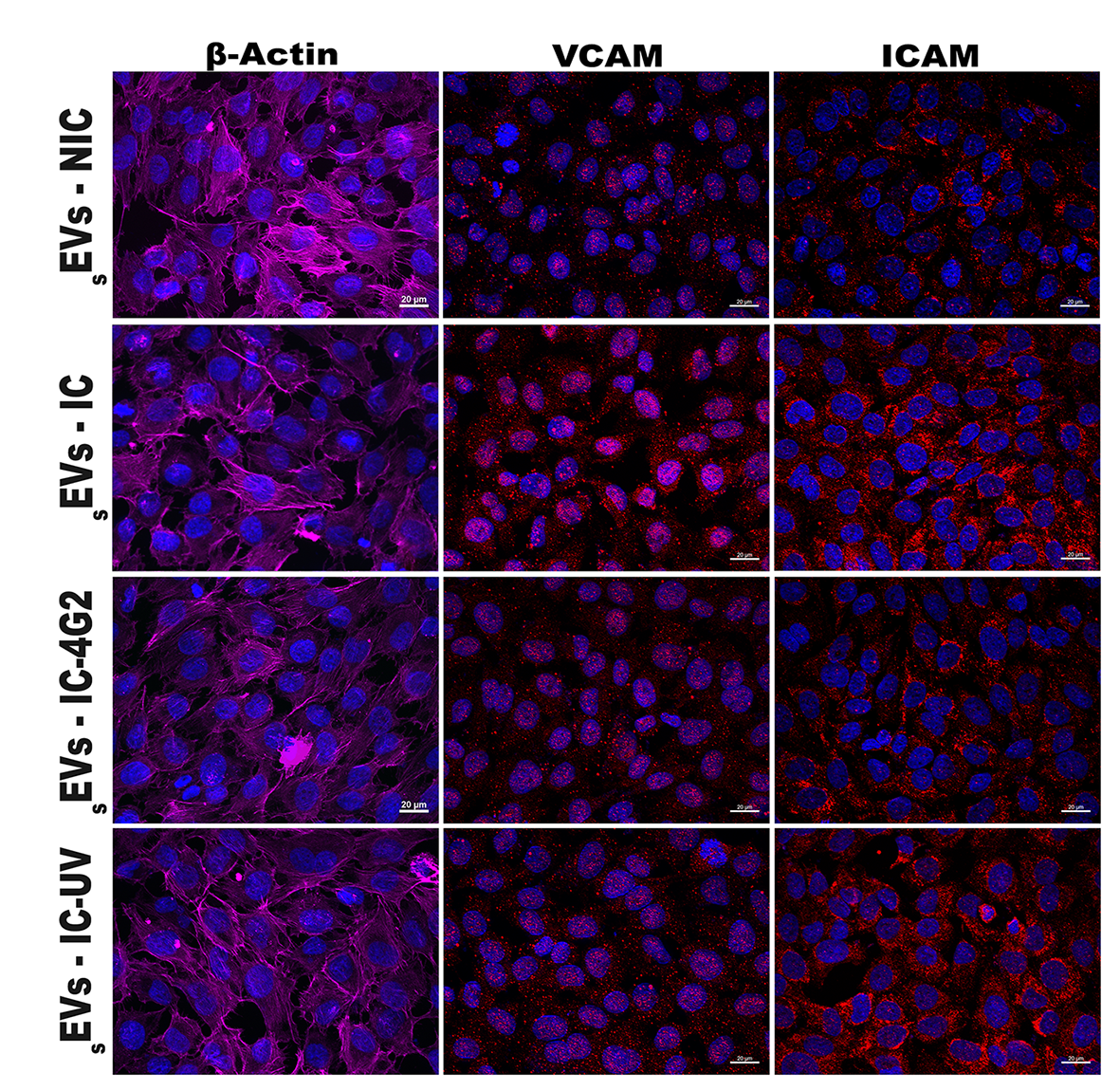

Supplement: S5 Fig — Prior infection, no morphological changes were evident in the EC; actin filaments remained stable, and cells seemed polygonal or elongated. The monolayer was confluent; however, some spaces were especially evident in the monolayer treated with sEVs-IC, which might be due to cell renewal processes, without being possible to rule out if this is an effect of the sEVs. Pre-treatment of the EC with the sEVs-IC caused cell activation that was more evident when cells were marked with the ICAM antibody. (TIF) [file pone.0310735.s005.tif]
